# Cellular and transcriptome signatures unveiled by single-cell RNA-Seq following *ex vivo* infection of murine splenocytes with *Borrelia burgdorferi*

**DOI:** 10.3389/fimmu.2023.1296580

**Published:** 2023-12-08

**Authors:** Venkatesh Kumaresan, Taylor MacMackin Ingle, Nathan Kilgore, Guoquan Zhang, Brian P. Hermann, Janakiram Seshu

**Affiliations:** ^1^ Department of Molecular Microbiology and Immunology, The University of Texas at San Antonio, San Antonio, TX, United States; ^2^ South Texas Center for Emerging Infectious Diseases, The University of Texas at San Antonio, San Antonio, TX, United States; ^3^ Department of Neuroscience, Developmental and Regenerative Biology, The University of Texas at San Antonio, San Antonio, TX, United States

**Keywords:** single-cell RNA-Seq, lyme disease, myeloid immune response, neutrophil apoptosis, complement, cytokines

## Abstract

**Introduction:**

Lyme disease, the most common tick-borne infectious disease in the US, is caused by a spirochetal pathogen *Borrelia burgdorferi* (*Bb*). Distinct host responses are observed in susceptible and resistant strains of inbred of mice following infection with *Bb* reflecting a subset of inflammatory responses observed in human Lyme disease. The advent of post-genomic methodologies and genomic data sets enables dissecting the host responses to advance therapeutic options for limiting the pathogen transmission and/or treatment of Lyme disease.

**Methods:**

In this study, we used single-cell RNA-Seq analysis in conjunction with mouse genomics exploiting GFP-expressing *Bb* to sort GFP+ splenocytes and GFP− bystander cells to uncover novel molecular and cellular signatures that contribute to early stages of immune responses against *Bb*.

**Results:**

These data decoded the heterogeneity of splenic neutrophils, macrophages, NK cells, B cells, and T cells in C3H/HeN mice in response to *Bb* infection. Increased mRNA abundance of apoptosis-related genes was observed in neutrophils and macrophages clustered from GFP+ splenocytes. Moreover, complement-mediated phagocytosis-related genes such as C1q and Ficolin were elevated in an inflammatory macrophage subset, suggesting upregulation of these genes during the interaction of macrophages with *Bb*-infected neutrophils. In addition, the role of DUSP1 in regulating the expression of Casp3 and pro-inflammatory cytokines Cxcl1, Cxcl2, Il1b, and Ccl5 in *Bb*-infected neutrophils were identified.

**Discussion:**

These findings serve as a growing catalog of cell phenotypes/biomarkers among murine splenocytes that can be exploited for limiting spirochetal burden to limit the transmission of the agent of Lyme disease to humans via reservoir hosts.

## Introduction

Lyme disease is the most common tick-borne disease with 40,000 confirmed cases, and more than 450,000 cases are estimated to occur in the US each year according to Centers for Disease Control and Prevention (CDC, Atlanta) (1). *Borrelia burgdorferi (Bb)*, the agent of Lyme disease, is a spirochete acquired by *Ixodes scapularis* ticks from a variety of small mammals that serve as reservoir hosts, most notably the white-footed mouse (*Peromyscus leucopus)* (2). Transmission to naive incidental vertebrate hosts, such as humans and dogs, occurs following the bite of infected ticks. Typical symptoms in humans include fever, headache, fatigue, and a characteristic skin rash called erythema migrans. If left untreated, *Bb* can disseminate to the joints, heart, and the nervous system resulting in manifestations of arthritis, carditis, and neuroborreliosis (3). Doxycycline is the antibiotic of choice. However, a subset of Lyme disease patients manifest a range of symptoms that are referred to as post-treatment Lyme disease syndrome (PTLDS) (4). The molecular mechanisms that lead to PTLDS are unclear, although there is evidence of subcellular components of *Bb* serving as immunogenic stimuli to sustain host inflammatory response in select tissues such as the joints (5, 6). Currently, there are no vaccines available for the prevention of Lyme disease in humans, although there are several candidate preparations at different stages of clinical trials in the US and in Europe (7, 8). Hence, strategies to limit the survival of *Bb* in the reservoir hosts to prevent or lower the kinetics of transmission via ticks to naive vertebrate hosts are in dire need to reduce the incidence of human Lyme disease.

Host–pathogen interactions play a key role in the dissemination, survival, and pathogenic effects of *Bb* in reservoir and incidental hosts (9, 10). Pathogen-specific components such as the cell envelope comprised of an outer membrane with a rich constellation of lipoproteins, a unique peptidoglycan, periplasmic flagella contributing to its shape and motility, and an inner membrane with several transport proteins enabling acquisition of host-derived nutrients mediate interactions with the soluble and cellular effectors of the hosts resulting in survival or clearance of *Bb* (11). Additionally, cytosolic proteins and enzymes modulate the pathogen metabolism in response to environmental signals and nutrients available for survival of *Bb* within different host milieu. The structure, function, and regulation of expression of components associated with the envelope and cytosol also enable the spirochetes to modulate the host innate and adaptive immune responses that either favor or limit survival of *Bb* (12). Therefore, targeting one or more mediators of the host response can be exploited to circumvent the survival of *Bb* in different hosts as an anti-virulence strategy to prevent Lyme disease.

Numerous studies on host–pathogen interactions exploited animal models of infection. Primarily, different inbred strains of mice, outbred white-footed mouse (*Peromyscus leucopus*), and primates have been used to delineate the host response against *Bb* infection with caveats to each model in terms of their relevance to human Lyme disease. Generally, mice infected via needle or tick challenge results in persistent infection in skin and dissemination of *Bb* to lymph nodes, spleen, bladder, heart, and joints from the site of challenge (13, 14). Differences in the host response to *Bb* infection between inbred strains of mice have provided key insights on the kinetics of inflammatory responses associated with Lyme borreliosis. Notably, factors influencing Lyme arthritis have been investigated by comparing C3H/HeN mice, which are genetically predisposed to developing severe arthritis upon *Bb* infection compared to relatively mild manifestations in C57BL/6 mice (15). Both gross and histopathological changes in the joints of C3H/HeN mice have been attributed to differences in the levels of Type1 interferon response and its regulatory effects on tissue repair genes between C3H/HeN and C57BL/6 mice (16). Strain variations in *Bb* has also been shown to play a role in the severity of manifestations in mouse models of infection (17). Moreover, genetic variability among the major surface exposed lipoproteins that mediate interactions between the pathogen and host determinants also contribute to differences in dissemination and tissue tropism (18–21). Additionally, the ability to genetically manipulate *Bb* to express green-fluorescent protein (GFP) or bioluminescent markers has provided tools that enable visualization and tracking of spirochetes interacting with host cells *in situ* and in real time (11, 22, 23).

Recently, it was shown that the upregulation of PD-1 and its ligand PD-L1 on CD4+ T cells and antigen-presenting cells, respectively, influenced the activation of T-cell populations in the joints without impacting *Bb* clearance (24). A quiescent immune response characterized by host complement, interferon alpha (IFN-α), tumor necrosis factor α (TNFα)−driven signaling, and inflammatory response was associated with resistance to *Bb* infection in the white-footed mouse in comparison to C3H/HeN mice (25, 26). This suggested that differences in host response and variations in *Bb* strains influence adaptive capabilities of Lyme disease pathogen (27). Primate models of infection have provided information on the efficacy of antimicrobial therapy, dissemination, and persistence of *Bb* in a mammalian host with significant DNA homology with humans. However, the lack of manifestations of neuroborreliosis has limited the utility of this model for understanding persistent infections in humans (28, 29). The presence of anti-phospholipid antibodies in the serum, or antibodies against borrelial peptidoglycan in the synovial fluid, of Lyme disease patients also adds to immunomodulatory effects of *Bb* that can be exploited both for diagnostic and as possible targets to abrogate the clinical manifestations of Lyme disease (6, 30). In addition, profiling the host response among various mammalian hosts to *Bb* infection provides a rich platform that can be exploited to strategically modulate the immune response in the reservoir hosts to block the natural life cycle of *Bb*.

Several studies have shown the role of innate and adaptive immunity during spirochetes infection in mammalian hosts. Among the innate immune cells, the role of macrophages and neutrophils during the early immune response against *Bb* have been investigated extensively (11). Complement-mediated phagocytosis is one of the well-studied protective mechanisms exhibited by macrophages to control *Bb* infection (31). Several prior studies have identified mechanisms adopted by *Bb* to evade the complement system either by direct interference with complement components or by binding the regulators of the host complement system (32). Apart from macrophages, apoptotic neutrophils also play an important role in controlling *Bb* infection. Hilliard et al. demonstrated that clearance of apoptotic neutrophils play a role in the resolution of inflammation during experimental Lyme arthritis through the activation of Peroxisome Proliferator Activated Receptor Gamma (PPAR-γ) (33). Many of these studies employed bulk cell RNA-Seq analysis to determine overall gene expression levels from an admixture of different cell types with both infected and uninfected bystander cells contributing to transcriptome profiles. It is likely that not all mammalian cells treated with bacterial pathogens are infected even under optimized *in vitro* conditions with different multiplicity of infection potentially leading to transcriptomes of bystander cells dampening or enhancing the overall response. Moreover, there is limited information available on the infected-cell-specific immune response against *Bb* infection, which is critical to understand the molecular changes leading to global or tissue-specific manifestations of Lyme disease.

The fast-developing field of single-cell technologies have brought mechanistic insights into the host–pathogen interactions that influence viral (34), bacterial (35), fungal (36), and parasitic infections (37). In this study, we performed single-cell RNA-Seq (scRNA-Seq) analysis with murine splenocytes infected with green fluorescent protein expressing *Bb* (GFP+*Bb*) and compared the cellular and molecular patterns between *Bb* infected (GFP-positive) and bystander (GFP-negative) splenocytes. Since *Bb* is an extracellular pathogen that resides primarily within the extracellular matrices of the host tissues, for purposes of this study, the GFP+ splenocytes are considered as “infected” and are represented by splenocytes that are either interacting with intact or have ingested/processed GFP-expressing spirochetes. Our study revealed novel information on the cellular and molecular changes mediated by *Bb* on the subset of macrophages and neutrophils that are specifically involved in the early stages of *Bb* infection.

## Materials and methods

### Mice and ethics statement

The animal facilities at The University of Texas at San Antonio (UTSA) are part of Laboratory Animal Resources Center (LARC), which is an Association for Assessment and Accreditation of Laboratory Animal Care (AAALAC) International Accredited Unit. Female C3H/HeN mice 6–8 weeks old (Charles River Laboratories, Wilmington, MA) were used in this study. All animal experiments were conducted following National Institutes of Health (NIH) guidelines for the housing and care of laboratory animals and in accordance with protocols approved by the Institutional Animal Care and Use Committee (protocol number MU071) of UTSA. Based on these guidelines, the general condition and behavior of the animals were monitored by trained laboratory and LARC staff daily, and methods to minimized pain and discomfort were adopted as needed in this study.

### Bacterial strain and growth conditions

A low passage infectious clonal isolate of GFP-expressing *Borrelia burgdorferi* B31-A3 (GFP+*Bb*) or wild-type *Borrelia burgdorferi* strain B31-A3 (WT-*Bb*) was propagated at 32°C in liquid Barbour–Stoenner–Kelly (BSK II, pH7.6) medium supplemented with 6% heat-inactivated rabbit serum (Pel-Freez Biologicals, Rogers, AR) with appropriate antibiotics (Sigma-Aldrich, St. Louis, MO) as previously described (22, 38–43). Once the cultures reached a density between 1 and 2×10^7^ spirochetes/ml, viable spirochetes were enumerated by dark field microscopy and used to infect murine splenocytes.

### Isolation of lipoproteins from *Bb*

Lipoproteins were extracted from the spirochetes as previously described (44, 45). Briefly, 1×10^9^ *Bb* was solubilized in 1 mL of phosphate-buffered saline (PBS) pH 7.4 containing 1% Triton® X-114 (TX-114) by gentle rocking at 4°C overnight. The TX-114 insoluble material was removed by two centrifugations at 15,000×*g* at 4°C for 15 min. The supernatant was transferred to a sterile tube and incubated at 37°C for 15 min. Then, the mixture was centrifuged at 15,000×*g* for 15 min at room temperature (RT). The top aqueous phase was transferred to a new tube and re-extracted one more time with 1% TX-114 as described. The bottom detergent phase was washed with 1 mL PBS pH 7.4 three times. The final detergent phase proteins were precipitated by adding 10-fold volume of ice-cold acetone, precipitates were collected by centrifugation at 15,000×*g* at 4°C for 30 min, acetone was removed by drying, and proteins were resuspended in PBS and stored at −20°C until further use. Lipoproteins were analyzed by Sodium Dodecyl Sulfate-Polyacrylamide Gel Electrophoresis (SDS-PAGE) and quantified using the bicinchoninic acid (BCA) assay kit (Thermo Scientific).

### Splenocyte isolation and infection

**Figure 1:**
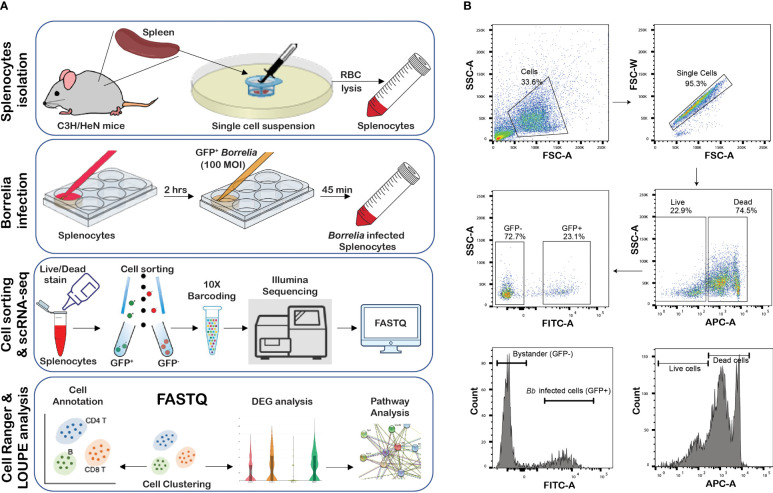
Infection of splenocytes with GFP+ *Bb.* **(A)** Overview of the study, **(B)** flow cytometry analysis of splenocytes infected with GFP+*Bb*. Total splenocytes were screened for single cells and further subjected to Live/Dead stain analysis, where APC^hi^ indicates dead cells, while APC^-^indicate live cells. Live, FITC^hi^ cells were designated as GFP+ (*Bb* infected cells), and FITC^neg^ cells were categorized as GFP− (bystander cells).

Spleens were removed from euthanized mice (*n*=5), homogenized using a 70-µm cell strainer, and single-cell suspension was then filtered through a 40-µm cell strainer. Splenocytes were pelleted by centrifugation at 500×*g* for 5 min. Red blood cells (RBCs) were lysed by resuspending the splenocytes in 5 mL of 0.14M ammonium chloride solution for 5 min at room temperature. RBC lysis was stopped by adding 10 mL of R10 media (RPMI media supplemented with 10% Fetal Bovine Serum (FBS)) and centrifuged at 500×*g* for 5 min and resuspended in R10 media at 1×10^6^/mL concentration. Furthermore, the cells were plated in six-well plates and allowed to adhere for 2 h. Non-adherent cells were removed, and the adherent cells were infected with GFP+*Bb* at a multiplicity of infection (MOI) of 1:1. Overall study design is explained in **Figure 1A**.

### Flow cytometry assisted cell sorting and single-cell RNA-Seq analysis

After 45 min of exposure to GFP+*Bb*, the splenocytes were scraped and labeled with Zombie R685^TM^ Fixable Viability Kit to determine live/dead cells (Biolegend, CA). Dead splenocytes (APC^hi^) were sorted out using flow-assisted cell sorter (BD FACS Diva-based cell-sorter), and the splenocytes were sorted into GFP+ (infected with GFP+*Bb*) and GFP− splenocytes as shown in **Figure 1B**. 10× Genomics single-cell libraries were generated by the University of Texas at San Antonio (UTSA) Genomics Core. Cell suspensions were loaded into 10× Genomics microfluidics chips and onto the 10× chromium to capture single cells in gel beads-in-emulsion (GEMs) reactions. We targeted collection of 10,000 cells/sample from two independent experiments, each using splenocytes collected from five mice, and followed the manufacturer’s recommendations for the 3′ Gene Expression v3 kit followed by Illumina sequencing of each library. Using the Cell Ranger analysis tools from the 10× Genomics cloud (https://cloud.10xgenomics.com/cloud-analysis), FASTQ files obtained from the infected and bystander samples from each individual experiment were individually processed, and the cell clusters were determined. Briefly, Cell Ranger count analysis was performed using the GRCm38 (mm10) mouse genome reference with default parameters for each infected and bystander sample from each experiment. Outputs from all four samples (infected and bystander) from both experiments were merged using CellRanger aggr (10× Genomics) based on mapped read count normalization and produce aggregated gene × cell barcode matrices and clustering models. Loupe Cell Browser v6.0.0 (10× Genomics) was used to visualize and analyze the data. To select only high-quality cells and eliminate multiplets, the aggregated data sets were limited to Unique Molecular Identifiers (UMIs) >500 and the genes per barcode >100. These settings were followed for all reclustering steps to avoid including multiplets and low-quality cells or empty droplets in the analysis. Cells expressing high proportions of mitochondrial genes (>5%) were excluded.

scRNA-Seq data sets were generated from two independent biological replicates (experiments 1 and 2) performed with different sets of mice, bacterial cultures, sorting, and scRNA-Seq analysis under similar experimental conditions. Both experiments revealed similar cell cluster patterns reflecting comparable cell populations from splenocytes in each experiment. Comparative transcriptome analysis between *Bb* infected (GFP+) and bystander (GFP−) cells revealed several differentially expressed genes (DEGs) that were significantly different in experiment 1. However, in experiment 2, the same DEGs in experiment 1 were identified although not with the same levels of significance (p<0.05) except for CXCL2 (significantly different between infected and bystander cells). We therefore validated 12 DEGs that were significantly different between infected and bystander cells from experiment 1 using methods to determine mRNA abundance, protein abundance, and flow cytometry analyses using commercially available reagents. The overall scRNA-Seq analysis workflow is described in **Supplementary Figure S1**, and data shown and validated were based on DEGs from experiment 1. All raw data files are deposited in NIH GEO under the accession number GSE243126.

### Protein network analysis

To understand the key pathways that were differentially activated between infected and bystander splenocytes and to analyze the interactions between the selected DEGs, protein network analysis was performed using the STRING database (https://stringdb.org/cgi/input?sessionId=byi0hUiTkgmG&input_page_show_search=on). Briefly, the significant DEGs were identified and submitted to the STRING database via the Multiple Proteins option, and the proteins with significant differences were searched against the mouse genome database. Protein–protein interactions between the selected genes were analyzed using the mouse protein–protein interaction database from STRING online database version 11.5, and the interaction prediction was determined with high confidence (0.700). Additionally, functional ontology analysis was performed to identify key molecular functions that were common within the selected genes. Furthermore, the interactions between the selected genes involved in specific pathways were also predicted.

### Isolation of bone-marrow-derived neutrophils

Neutrophils were isolated from the bone marrow of C3H/HeN mice using negative selection magnetic beads technique (46). Briefly, five C3H/HeN mice were euthanized using CO_2_ exposure and followed by cardiac puncture as the second method of euthanasia. Using sterile techniques, the femur and tibia were collected, and the bone marrow cells were flushed with sterile R10 media (RPMI media supplemented with 10% FBS), using a 25-gauge needle and centrifuged at 500×*g* for 5 min at 4°C (47). Cell pellet was resuspended in 4 mL of 0.14M ammonium chloride solution to lyse RBCs. After a 5-min incubation at RT, RBC lysis was stopped by adding 8 mL of fresh R10, and the contents were passed through the 70-µm cell strainer and centrifuged at 500×*g* for 5 min at 4°C. The cell pellet was washed with cold PBS and subjected to magnetic separation using a neutrophil isolation kit (Miltenyi Biotech GmBH, Germany) in accordance with the manufacturer’s instructions. Cell viability was determined by Trypan blue staining and the cells enumerated using a hemocytometer, and the purity of the neutrophils was assessed to be >98% for each assay by flow cytometry using APC-conjugated anti-mouse Ly6G antibody (BioLegend). Freshly isolated BMNs were infected with 100 MOI of WT-*Bb* resuspended in 100 µL of BSKII media pH 6.8 (48). BMN treated with only BSKII media was maintained as a control. After 1, 4, or 24 h post-infection, media were removed and cell washed with cold PBS and used for RNA isolation, protein extraction, or flow cytometry as required.

### Generation of bone-marrow-derived macrophages

As mentioned earlier, bone marrow cells were collected from five C3H/HeN mice, pooled and centrifuged at 500×*g* for 5 min at 4°C. Cell pellet was resuspended in 4 mL of 0.14M ammonium chloride solution to lyse RBC. After 5 min incubation at RT, RBC lysis was stopped by adding 8 mL of fresh R10 and centrifuged at 500×*g* for 5 min at 4°C. Cells were resuspended in 40 mL of R10 + 15% LCCM and passed through a 70-µM cell strainer. A total of 10 mL of cell suspension was plated in each Petri dish and incubated at 37°C/5%CO_2_ in a cell culture incubator. After 4 days, 50% of the media was replaced with fresh R10 media and incubated at 37°C. After 8 days of initial plating, the culture media was removed, and cells were washed twice with 10 mL of cold PBS. To detach the adherent cells that have been differentiated into bone-marrow-derived macrophages (BMDMs), 2 mL of Accutase™ Cell Dissociation Reagent (STEMCELL Technologies) was added and incubated at 37°C for 20 min. Plates were gently tapped to detach BMDMs, and the cell suspension was supplemented with 4 mL of fresh R10 media and centrifuged at 500×*g* for 5 min. The cells were resuspended in fresh R10 media without Pen/Strep and seeded in U-bottom 96-well plates (Corning) at 1×10^5^ BMDMs per well. After overnight incubation, the cells were infected with 100 MOI of WT-*Bb* resuspended in 100 µL of BSKII media pH 7.6. BMDM treated with only BSKII media was maintained as control. After 1 hpi, 4 hpi, or 24 hpi, media were removed and washed with cold PBS, and the cells were pooled and used for RNA isolation, protein extraction, or flow cytometry as needed.

### RNA isolation and quantitative reverse transcription PCR analysis

BMNs and BMDMs were infected with WT-*Bb* at an MOI of 1 in 96-well round-bottom plates at 1×10^5^ cells per well, washed and centrifuged at 500×*g* for 5 min. The supernatant was discarded, and the cell pellets with and without *Bb* infection from multiple wells were pooled, and RNA was isolated. At 1 hpi and 24 hpi, RNA was isolated using RNeasy kit (Qiagen, Germany) according to the manufacturer’s protocol. A constant amount of RNA (1 µg per sample) was reverse transcribed into cDNA using Advanced iScript cDNA synthesis kit (BioRad). cDNA was then amplified using murine gene-specific primers (**Supplementary Table S1**), and the primers for Actb gene were used as normalization control. Real-time quantitative PCR was performed by StepOne Plus Real-Time PCR system (Applied Biosystems) using default PCR program. Results are represented as fold difference using 2^−ΔΔct^ formula compared to the internal and the uninfected controls.

### Co-culture of BMDM with *Bb*-infected BMNs

To determine the role of *Bb*-infected neutrophils in the upregulation of genes encoding complement components in macrophages, we infected BMNs with *Bb* for 16 h based on the observation that Casp3 upregulation in BMNs was significant at 16 hpi. Furthermore, *Bb*-infected BMNs were treated with BMDM at BMN:BMDM ratio of 1:1 and 1:10. At 2 hpi, the total cell suspension was pelleted, and RNA was isolated as described above. Using the cDNA synthesized from these RNA samples, the mRNA abundance of the complement genes C1qa, C1qb, C1qc, and Fcna were determined, and the data were normalized using Actb gene.

### Validation of frequency of infected neutrophil and macrophage cell population by flow cytometry

To validate the number of infected neutrophils and macrophages among splenocytes during *Bb* infection observed in scRNA-Seq analysis, spleens were isolated from three C3H/HeN mice and splenocytes were purified by homogenizing the spleen in a cell strainer (40 µM), RBC was depleted by ACK lysing solution (Lonza) at room temperature for 5 min and centrifuged at 500×*g* for 5 min at 4°C. The cell pellet was resuspended in RPMI–10% FBS, plated in a non-treated six-well plate and infected with GFP+*Bb* at 1:1 ratio. After 45 min of incubation at 37°C, cells were centrifuged at 500×*g* for 5 min, and the cells were fixed using 200 µL of Cell Fixation Buffer (Biolegend) for 20 min in the dark. Cells were washed with 1 mL of Cell Staining Buffer (Biolegend) to remove unbound fixative. To avoid non-specific binding of antibodies, 1 µg of CD16/CD32 antibody dissolved in 100 µL of cell staining buffer was added to 5×10^6^ cells and incubated for 10 min on ice in the dark. One microgram of anti-CD45-PE, anti-CD11b-PE-cy7, anti-Ly6G-APC-Cy7, and anti-F4/80-APC (Biolegend) was dissolved in 100 µL of cell staining buffer and added to each cell suspension and incubated on ice for 20 min in the dark. Finally, the cells were washed with 1mL of cell staining buffer twice, and flow cytometry analysis was performed using LSRII (BD bioscience); 300,000 events were acquired and analyzed using FlowJo software.

To determine Casp3 activation in BMNs during *Bb* infection or following exposure to borrelial lipoproteins (*Bb*Lp), live BMNs were stained with YO-PRO^TM^-1 Iodide (YP-1), a cell-permeable dye that binds with active Casp3 (Thermo Fisher, USA). The working solution of YP-1 was prepared as 0.1 µM YP-1 stain in sterile PBS, according to the manufacturer’s protocol. At 1 hpi and 24 hpi, BMNs were pelleted and resuspended in 200 µL of YP-1 stain and incubated on ice for 30 min. YP-1-positive cells were identified by flow cytometry analysis using LSRII (BD bioscience), and the results were analyzed using FlowJo software.

### Cytokine quantification

To quantify the cytokines Cxcl1, Cxcl2, Ccl5, and Il1b in the culture supernatant of BMN and BMDM, with or without *Bb* infection, ELISA was performed using Mouse CXCL1/KC, CXCL2/MIP-2, Ccl5/RANTES, and Il1b/Il-1F2 DuoSet ELISA kits (R&D Systems, Minneapolis, MN, USA), following the manufacturer’s protocols. Samples were diluted accordingly to measure the OD within the linear range of the standards for each of the cytokine at 450/570 nm using a Spark 10M (Tecan) multimode plate reader and recorded using Spark software.

### Statistical analysis

Graphs were prepared, and unpaired *t*-test was performed using GraphPad Prism 7.0. Statistical differences between groups were reported to be significant when the *p*-value was ≤0.05. Data are presented as mean ± standard error of mean (SEM).

## Results

### scRNA-Seq analysis identifies the cellular diversity in murine splenocytes

**Figure 2:**
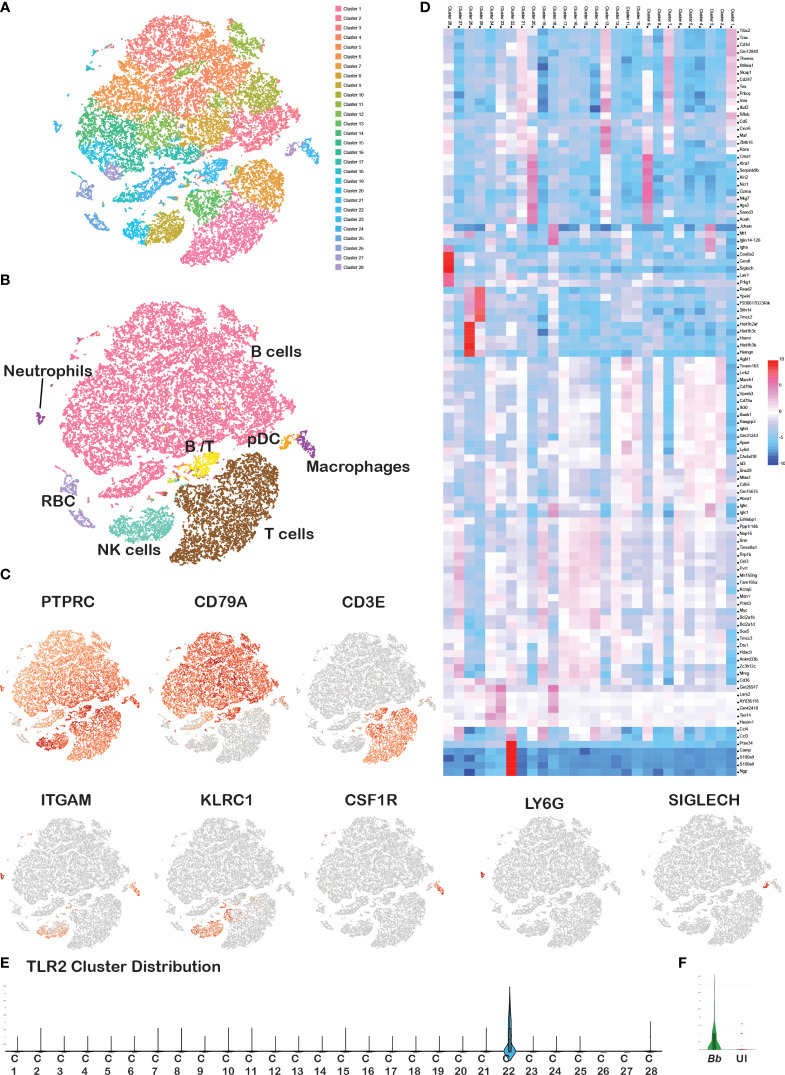
Cell cluster analysis of total splenocytes. Barcodes obtained from *Bb*-infected and bystander splenocytes from two experiments were integrated together to identify the cell clusters. **(A)** tSNE plot showing 28 cell clusters among the total splenocytes, **(B)** annotated clusters of total splenocytes, **(C)** expression of cell-specific marker genes in total splenocytes, **(D)** heatmap of DEGs between the overall 28 clusters. Scale represents the differential expression of genes (upregulated—red, downregulated—blue) as log2 fold changes where the cluster numbers are mentioned on the top of each column and the gene names at the right of each row. Violin plots showing the Log2 expression level of TLR2 gene **(E)** in overall 28 clusters, and **(F)** in Ly6G+ barcodes compared between *Bb*-infected and bystander.

Splenocytes from 6- to 8-week-old C3H/HeN mice were infected with GFP+ *Bb* for 45 min, and flow-assisted cell sorting was performed to segregate GFP+ cells as shown schematically in **Figure 1A**. Flow cytometry analysis indicated that 23% of sorted splenocytes were GFP+ve and 74.7% cells were GFP−ve (**Figure 1B**). The sorted GFP+ve (infected) and GFP−ve (bystander) splenocytes from two independent experiments were used for scRNA-Seq analyses, and splenocytes fell into 28 clusters (**Figure 2A**). Based on the established canonical markers of various lymphocytes and myeloid cells, and through interrogation of murine gene expression atlas, we assigned putative biological identities to the clusters as CD3+ T cells (clusters 1, 7, 12), CD79a+ B cells (clusters 2, 3, 4, 5, 6, 8, 11, 13, 14, and 16) and KLRC+ natural killer cells (cluster 9) (**Figure 2B**). Furthermore, cluster 22 comprised of most of the myeloid cells such as neutrophils (CD11b+Ly6G+), macrophages (CD11b+F4/80+), and dendritic cells (CD11c+CD8a+) (**Figure 2C**). Cluster 10 exhibited low level CD45+ cells, indicating that this cell population is most likely to be erythrocytes. Heatmap analysis showed the difference in the gene expression pattern of the 28 clusters identified from the total splenocytes with clusters 9 (NK cells), 26 (erythrocytes), and 22 (myeloid cells) had unique DEGs compared to other clusters (**Figure 2D**).

A comparison of tSNE plots between infected and bystander cells showed a similar cluster distribution pattern with no apparent differences between the two samples (**Supplementary Figure S2A**). Cell count analysis of each immune cell population of splenocytes revealed that B (65%) and T (25%) lymphocytes occupied the largest proportion, followed by the NK cells (5%) and myeloid cells (5%) (**Supplementary Figure 2B**), which is consistent with the traditional cell profiling of spleen (49). A comparison of individual immune cell populations with PTPRC+ bar codes from tSNE plots from both experiments showed that the number of neutrophils and macrophages were significantly higher in *Bb*-infected splenocytes compared to the bystander cells (**Supplementary Figure 2B**). Further validation of splenocytes infected with *Bb*-GFP using flow cytometry revealed a significant difference in the number of GFP+ neutrophils (CD11b+ Ly6G+) and macrophages (CD11b+F4/80+) consistent with the profile observed via scRNA-Seq analysis (**Supplementary Figure S3**). However, there were no significant differences in percentage of other cell populations between the infected and bystander splenocytes. TLR2, which plays an important role during *Bb* infection, was expressed only in cluster 22 (myeloid population) among the 16 clusters identified in scRNA-Seq analysis (**Figure 2E**). Among the myeloid cells, TLR2 was upregulated in Ly6G+ neutrophils population among *Bb*-infected splenocyte compared to uninfected bystander splenocytes (**Figure 2F**).

To characterize the subsets of each immune cell population and to analyze the DEGs between infected and bystander population, we classified and reclustered these cell populations independently. First, cells expressing pan leukocyte marker, CD45 (PTPRC>0), were used for the further analysis of various subsets of leukocytes and evaluated based on known markers such as CD79a (B cells), CD3e (T cells), KLRC1 (NK cells), ITGAM (myeloid), CSF1R (monocytes/macrophages), SIGLECH (eosinophils), and Ly6G (neutrophils) as displayed in **Figure 2C**. All genes discussed along with their corresponding protein IDs and full names of those genes were tabulated in **Supplementary Table S2**. The list of DEGs upregulated in each cluster compared to all other clusters is tabulated in **Supplementary Table S3**. DEGs that were significantly different between infected and bystander cells from experiment 1 were validated using qRT-PCR, cytokine enzyme-linked immunosorbent assay (ELISA), and flow cytometric analysis as described below.

### Gene expression heterogeneity in splenic neutrophils

**Figure 3:**
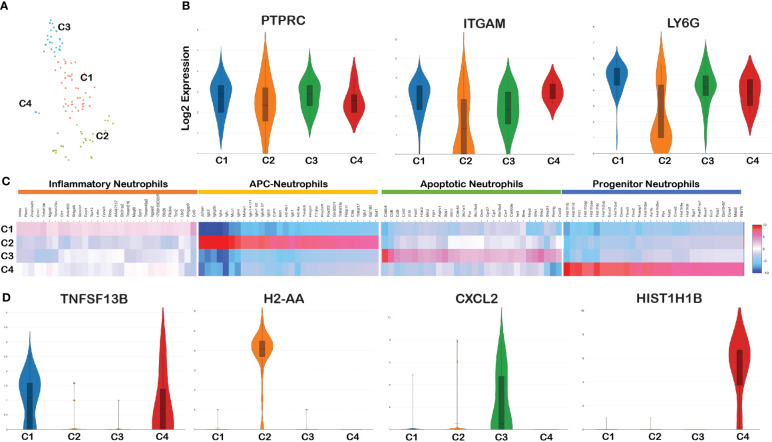
Cell cluster analysis of neutrophils. Barcodes filtered for PTPRC+ CD11b+ Ly6G+ were defined as neutrophils. **(A)** tSNE plot of neutrophils showing four clusters marked as C1, C2, C3, and C4. **(B)** Violin plots showing the log2 expression and cellular distribution of the selected neutrophil specific marker genes, and the gene names are mentioned at the top of each plot. **(C)** Heatmap showing the DEGs among the four clusters of neutrophils, and the putative names of each cluster were mentioned over the respective blocks. **(D)** Violin plots showing the log2 expression and cellular distribution of the selected cluster-specific genes.

CD11b^+^Ly6G^+^ markers are the most widely used markers to distinguish neutrophils from the remaining myeloid cells (50). Therefore, we annotated cells positive for PTPRC (CD45), ITGAM (CD11b), and Ly6G genes as neutrophils. To delineate the neutrophil heterogeneity and to identify the subsets of neutrophils, we filtered CD45+Cd11b+ Ly6G+ barcodes and re-clustered this population. The tSNE plot of the re-clustered neutrophils revealed four different sub-clusters of neutrophils (**Figure 3A**).

Violin plot analysis showed no significant changes in the expression of three genes (PTPRC, ITGAM, and Ly6G) that define neutrophil populations between the four clusters (**Figure 3B**). The list of DEGs upregulated in each neutrophil cluster is tabulated in **Supplementary Table 4A**. We compared the gene expression pattern of these four clusters and identified the unique genes expressed in each cluster. Clusters 1 and 4 expressed inflammation related genes such as Tnfsf13b (51), Plscr1 (52), and Ankrd22 (53) and hence termed as inflammatory neutrophils (54). Along with the expression of inflammatory genes, cluster 4 is characterized by high levels of H1 linker histones such as Hist1H1B, Hist1H1D, Hist1H3c, and Nusap1. Since they express H1 linker histone genes, an epigenetic regulator of cell proliferation and differentiation (55), it is possible to annotate this cluster as progenitor neutrophils expressing DEGs involved in cell cycle/proliferation (56). Cluster 2 exhibited two different populations with high and low ITGAM and Ly6G expression pattern compared with the other three clusters (**Figure 3B**). DEG analysis between the four clusters indicated that cluster 2 was enriched with B-cell related genes such as CD79a, Iglc3, Igkc, and Jchain and antigen-presenting MHC-II molecules such as H2-Aa, H2-Ab1, H2-Eb1, H2-Eb2, and CD74 (**Figure 3C**). Because of the activation/expression of MHC-II and co-stimulatory molecules expressed in neutrophils among *Bb-*infected cells, this cluster most likely is involved in antigen presentation and are classified as antigen-presenting neutrophils (57). Cluster 3 uniquely expressed genes such as Retnlg, Cxcl2, and Il1f9 and metalloprotease encoding genes such as Mmp8 and Mmp9 that are involved in macrophage polarization (58) and surface-expressed macrophage inducible proteins, Clec4d/Clec4e (59). The key DEG from each cluster is shown in **Figure 3D**. Thus, the spatial transcriptome profiling of neutrophils in the mouse spleen revealed four individual clusters expressing unique gene expression profiles with the potential to drive the innate immune responses of the host against *Bb*.

### Differential gene expression profiles between infected and bystander neutrophils

**Figure 4:**
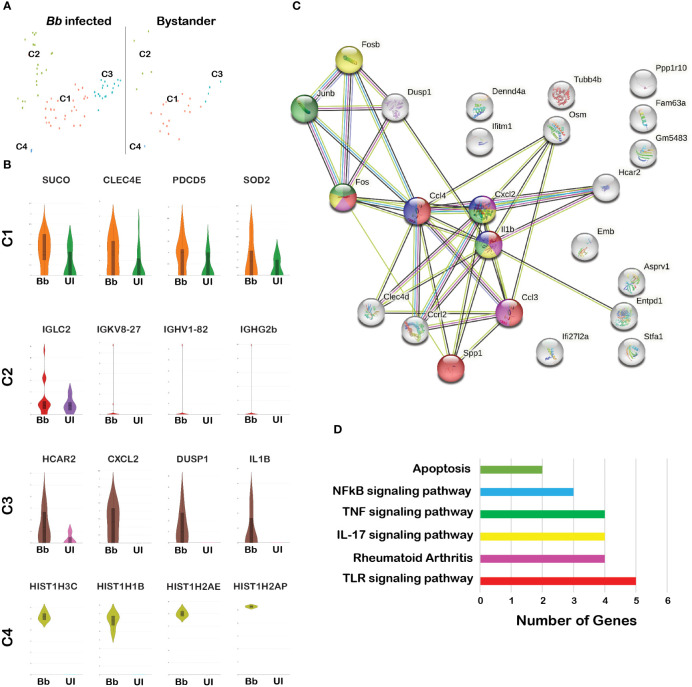
Comparison of the infected and bystander neutrophil clusters. **(A)** tSNE plot of *Bb*-infected and bystander neutrophils showing four clusters. **(B)** The violin plots showing the log2 expression (height) and cellular distribution (width) of the selected DEGs compared between the *Bb*-infected and bystander in each neutrophil cluster. **(C)** STRING-protein network showing the interaction of the selected DEGs identified from the *Bb*-infected cluster 3 neutrophils. **(D)** Bar graph showing the functional pathways enriched within the identified DEGs from the *Bb*-infected cluster 3 neutrophils, where each bar represents the number of genes enriched in each pathway. Colors of each circle **(C)** and bar **(D)** represent the corresponding functional pathway of each gene.

All four clusters of neutrophils were present in both infected and bystander populations (**Figure 4A**). A comparison of DEGs between infected and bystander cells in various clusters helped further to eluciate the modulatory effects of *Bb* **Supplementary Table 4b**. Among *Bb*-infected cells, Suco, Clec4e, Pdcd5, and Sod2 were upregulated in cluster 1, and immunoglobulin-related genes were upregulated in cluster 2 (**Figure 4B**). The Major DEGs upregulated in *Bb*-infected cluster 3 were Il1b, Hcar2, Zfp36, dual specific phosphatase 1 (DUSP1), Clec4d, and chemokines Ccl3, Ccl5, Cxcl1, Cxcl2, and Ccrl2 (**Figures 4B, C**). In cluster 4, mostly histone-related genes were upregulated in infected neutrophils (**Figure 4B**). The majority of DEGs expressed in *Bb*-infected cluster 3 were associated with pathways, indicating that these genes are functionally connected (**Figure 4C**). Several Kyoto Encyclopedia of Genes and Genomes (KEGG) pathways were identified based on the protein network analysis of DEGs expressed in cluster 3 of infected neutrophils such as IL-17 signaling pathway, Toll-like receptor (TLR) signaling pathway, nuclear factor kappa light chain enhancer of activated B cells (NF-κβ) signaling pathway, Tumor necrosis factor (TNF) signaling pathway, Mitogen‑activated protein kinase (MAPK) signaling pathway, and apoptosis (**Figure 4D**). Along with the profile of genes involved in apoptosis, cluster 3 was also enriched in signaling molecules such as macrophage-inducible proteins and metalloproteases, which are potentially involved in the neutrophil–macrophage chemotaxis in efferocytosis of apoptotic neutrophils by macrophages to combat infection and inflammatory responses (58–61). In *Bb*-infected cluster 2 (antigen-presenting neutrophils), immunoglobulin genes such as Iglc2, Igkv8-27, Ighv1-82, and Ighg2b were upregulated compared to the bystander cells, indicating that these neutrophils are likely involved in the activation of B and T cells following interactions with *Bb*. Further investigation is required to define the functional significance of this subset during *Bb* infection.

### *Bb* infection induces caspase 3 in bone-marrow-derived neutrophils

**Figure 5:**
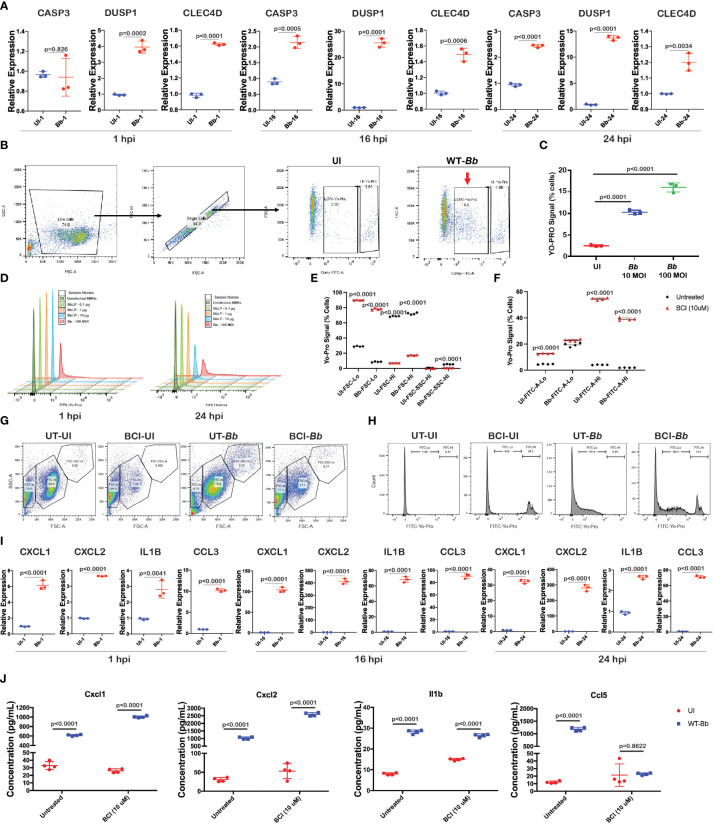
*Bb* induce pro-inflammatory cytokines and apoptosis in BMNs. **(A)** Gene expression analysis of selected genes using qRT-PCR analysis comparing uninfected and *Bb*-infected BMNs at 1,16, and 24 hpi represented as relative folds compared to the uninfected neutrophils at 1 hpi, **(B)** schematic representation of the gating used to determine the FITC (YO-PRO)-positive cells. **(C)** Percentage of cells positive for YO-PRO signals in BMNs infected with *Bb* (10 MOI) and *Bb* (100 MOI) compared to the uninfected control. **(D)** Histogram showing the pattern of YO-PRO signal in uninfected BMNs and BMNs treated with 0.1, 1, and 10 µg of *Bb*Lp and infected with *Bb* at 100 MOI, determined at 1 hpi (left) and 24 hpi (right). **(E)** Graph showing the distribution of FSC-lo, FSC-hi, and FSC-SSC-hi populations in BMNs during *Bb* infection with or without BCI treatment. **(F)** BMNs treated with or without BCI were infected with *Bb* and stained with YO-PRO, and flow cytometry analysis was performed. Graph showing the FITC-lo and FITC-hi population in BCI-treated or untreated BMNs with or without infection. **(G)** Dot plot showing the distribution of FSC-lo, FSC-hi, and FSC-SSC-hi populations in BMNs. **(H)** Histogram showing the FITC-lo and FITC-hi levels in BMNs. **(I)** Gene expression analysis of selected pro-inflammatory cytokines using qRT-PCR comparing uninfected and *Bb*-infected BMNs at 1 hpi, 16 hpi, and 24 hpi represented as relative folds compared to the uninfected neutrophils at 1 hpi. **(J)** Levels of cytokines Cxcl1, Cxcl2, Ccl5, and Il1b were determined by ELISA from the culture supernatant of BMNs infected with *Bb* (100 MOI) with or without BCI inhibitor treatment. BMNs infected with *Bb* at 100 MOI were stained with YO-PRO stain at 1 and 24 hpi, flow cytometry analysis was performed, and the signals were recorded using FITC channel. All qRT-PCR experiments were performed independently twice, and the data displayed are representative of one experiment showing the average values of at least three technical replicates. Data from second experiment are presented in **Supplementary Figure S4**. Error bar represents the standard deviation between the technical replicates. The p-values in each graph represents the significance of differences between the *Bb*-infected and bystander populations.

scRNA-Seq data revealed that DEGs involved in apoptosis are differentially expressed in *Bb-*infected neutrophils compared to bystander neutrophils (**Figures 4C, D**). To validate the mRNA abundance of genes encoding apoptosis effector caspase (CASP3) and inflammation-regulator genes (CLEC4D and DUSP1) (62, 63) during *Bb* infection in neutrophils, Quantitative Reverse Transcription PCR (qRT-PCR) analysis was performed (**Figure 5A**). Briefly, naive neutrophils isolated from bone marrows (BMNs) using magnetic separation were infected with WT-*Bb*, and RNA was isolated at 1 hpi, 16 hpi, and 24 hpi. Uninfected BMNs were used as controls, and the mRNA abundance of select genes was normalized using ß-actin gene as internal control. In *Bb*-infected BMNs, DUSP1 and CLEC4D showed consistent upregulation at 1 h, 16 h, and 24 h post-infection (hpi), while Casp3 was significantly upregulated at 16 hpi and 24 hpi (**Figure 5A**). These results indicated that apoptosis is activated in neutrophils via Casp3 activation, where DUSP1 and CLEC4D could regulate the Casp3 upregulation in the neutrophils during *Bb* infection.

To identify the involvement of Casp3-dependent apoptosis in neutrophils during *Bb* infection, BMNs were infected with *Bb* at 10 and 100 MOI and the Casp3 activity in BMNs was determined at 1 hpi using green-fluorescent YO-PRO™-1 (YP-1) Iodide staining, and flow cytometry was performed (**Figure 5B**). YO-PRO™-1 (YP-1) is a cell-impermeant dye that specifically binds to Casp3, used to identify Casp3-mediated apoptosis in cells. Compared to the uninfected BMNs, a significantly higher number of BMNs were positive for YP-1 stain in a dose-dependent manner (**Figure 5C**). To further understand the role of *Bb* Lipoproteins (*Bb*Lp) in the activation of Casp3 in BMNs, 1×10^6^ BMNs were infected with 10 µg, 1 µg, and 0.1 µg of purified *Bb*Lp or 100 MOI of *Bb* and Casp3 activity was determined at 1 hpi and 24 hpi. A total of 10 µg/ml *Bb*Lp induced significant activation of Casp3 at 24 hpi compared to lower doses, whereas no change in apoptosis was observed at 1 hpi. However, compared to *Bb*Lp, WT-*Bb* induced higher levels of Casp3 activation in BMNs both at 1 hpi and 24 hpi (**Figure 5D**).

To expand our understanding of the role of DUSP1 in regulating the Casp3 expression during *Bb* infection, BMNs were pretreated with BCI (DUSP1 inhibitor) for 1 h and then infected with WT-*Bb* for 24 h, stained with YP-1 followed by flow cytometry analysis. To compare the difference in granularity of neutrophils during *Bb* infection and determine the role of BCI in modulating the granularity, Forward versus side scatter (FSC-A vs. SSC-A) gating analysis was performed, and BMNs were grouped into three different clusters, FSC-lo, FSC-hi, and FSC-SSC-hi (**Figure 5G**). BCI-treated BMNs showed significantly higher FSC-Lo and lower FSC-hi compared to untreated BMNs irrespective of *Bb* infection (**Figure 5E**). *Bb* infection significantly increased the FSC-SSC-hi population compared to the uninfected BMNs, while BCI treatment reduced the FSC-SSC-hi population in *Bb*-infected BMNs. FSC indicates cell size, while SSC indicates cell granularity. Therefore, it is possible to conclude that *Bb* infection increases the granularity in neutrophils. Cell count analysis showed that *Bb* infection induced significantly higher YP-1-low population in BMNs compared to uninfected BMNs, whereas BCI-treated BMNs exhibit significantly increased YP-1-hi population than the untreated BMNs irrespective of *Bb* infection (**Figures 5F, H**). These observations showed that the inhibition of DUSP1 significantly alters the caspase 3 expression in BMNs with or without *Bb* infection.

### *Bb* infection stimulates pro-inflammatory cytokines in neutrophils

To validate the mRNA abundance of genes encoding pro-inflammatory cytokines (Cxcl1, Cxcl2, Ccl5, and Il1b) during *Bb* infection in neutrophils, qRT-PCR analysis was performed. Cxcl1, Cxcl2, Ccl5, and Il1b were upregulated in a time-dependent manner in *Bb*-infected BMNs compared to the corresponding uninfected BMNs (**Figure 5I** and **Supplementary Figure S4**). Furthermore, to understand the role of DUSP1 in controlling the cytokines expression in BMN during *Bb* infection, BMNs were treated with BCI (DUSP1 inhibitor) for 1 h at 37°C and then infected with WT-B31. Consistent with our mRNA abundance measurements, levels of Cxcl1, Cxcl2, Ccl5, and Il1b in culture supernatants collected at 24 hpi were significantly higher in *Bb*-infected BMNs compared to the uninfected BMNs (**Figure 5J**). Interestingly, Cxcl1 and Cxcl2 levels were significantly higher in BCI-treated *Bb*-infected BMNs compared to untreated *Bb*-infected BMNs. In contrast, Ccl5 level was significantly lower in BCI-treated *Bb*-infected BMNs compared to the untreated *Bb*-infected BMNs. However, the Il1b expression remained unchanged between BCI-treated and untreated BMNs infected with *Bb*. These observations indicated that *Bb* infection induces strong cytokine signaling in neutrophils, where DUSP1 plays a major role in regulating the Cxcl1/2 axis in neutrophils during *Bb* infection. The reduction in Ccl5 expression in BCI-treated *Bb*-infected BMNs indicates that DUSP1 is critical for the Ccl5 expression in BMNs during *Bb* infection.

### Gene expression heterogeneity in splenic macrophages

**Figure 6:**
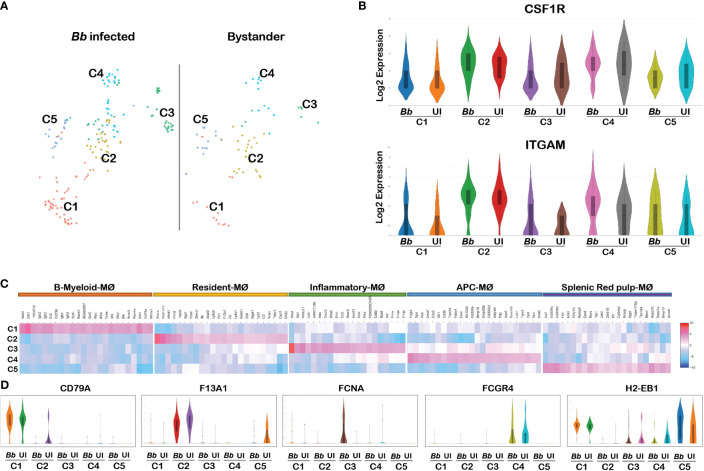
Cell cluster analysis of macrophages. Cells filtered for PTPRC+ ITGAM+ CSF1R+ were annotated as macrophages. **(A)** tSNE plot of macrophages showing five clusters marked as C1, C2, C3, C4, and C5. **(B)** Violin plots showing the log2 expression and cellular distribution of the macrophage-specific marker genes, and the gene names are mentioned at the top of each plot. Uniform expression of CSF1R and ITGAM genes in both infected (*Bb*) and bystander (UI) populations of each cluster. **(C)** Heatmap showing the DEGs among the four clusters of macrophages, and the putative names of each cluster were mentioned over the respective blocks. **(D)** Violin plots showing the log2 expression and cellular distribution of the selected cluster-specific genes between the infected (*Bb*) and bystander (UI) populations in each cluster.

Monocyte–macrophage differentiation is controlled by colony-stimulating factor (CSF1), and mice lacking this factor or the receptor, CSF1R, are profoundly macrophage deficient (64). CSF1 signaling is also required for the maintenance of the macrophage populations in adult mice (65). Therefore, we used CSF1R as a marker gene to classify the macrophage cluster among all cells that were positive for pan leukocyte marker, PTPRC. The CSF1R+ macrophage cluster occupied a major portion of cluster 22, comprising of two sub-clusters in t-SNE plot. Reclustering of PTPRC+CSF1R+ cells revealed five sub-clusters (**Figure 6A**). Violin plots of marker genes (ITGAM and CSF1R) revealed no significant changes in all five clusters (**Figure 6B**). Heatmap analysis showed that all five clusters expressed unique DEGs, indicative of transcriptionally discrete populations of cells (**Figure 6C**). The list of DEGs upregulated in each macrophage cluster is tabulated in **Supplementary Table S5A**.

Among the above six clusters, cluster 1 expressed both myeloid and B-cell-related genes, which are designated as B-myeloid macrophages (Cd79a, Ighm, and Igkc) (66). Cluster 2 expressed genes that are specific for resident macrophages (F13a1, Ly6c1, Ly6c2, Lyz2, and Fn1) (67). Cluster-specific gene expression analysis indicated that genes encoding complement component (C1qa, C1qb, C1qc, and Fcna), macrophage-inflammatory proteins (Ccl3 and tnf), neutrophil attractants (Cxcl1 and Cxcl2), and macrophage activation protein encoding genes (CSF1 and Acod1) were upregulated in cluster 3 (**Figure 6C**). The activation of complement and Ccl3 are key events in M1 polarization of macrophages; hence, we designated cluster 3 as inflammatory macrophages (68, 69). Cluster 5 expressed genes that are specific for splenic red-pulp macrophages (Adgre4, Treml4, Fcgr4, Fabp4, and Pecam1) (70). Cluster 4 expressed genes that are specific for macrophages that are involved in antigen presentation via class II MHC (H2-Aa, H2-Ab1, H2-Eb1, Cd74, and Ctss) (71). Cluster-specific expression of the selected genes are displayed in **Figure 6D**. These cluster-specific genes provide insights into the effects of *Bb* infection on splenocytes during early stages of infection.

### Differential gene expression profiles between infected and bystander macrophages

**Figure 7:**
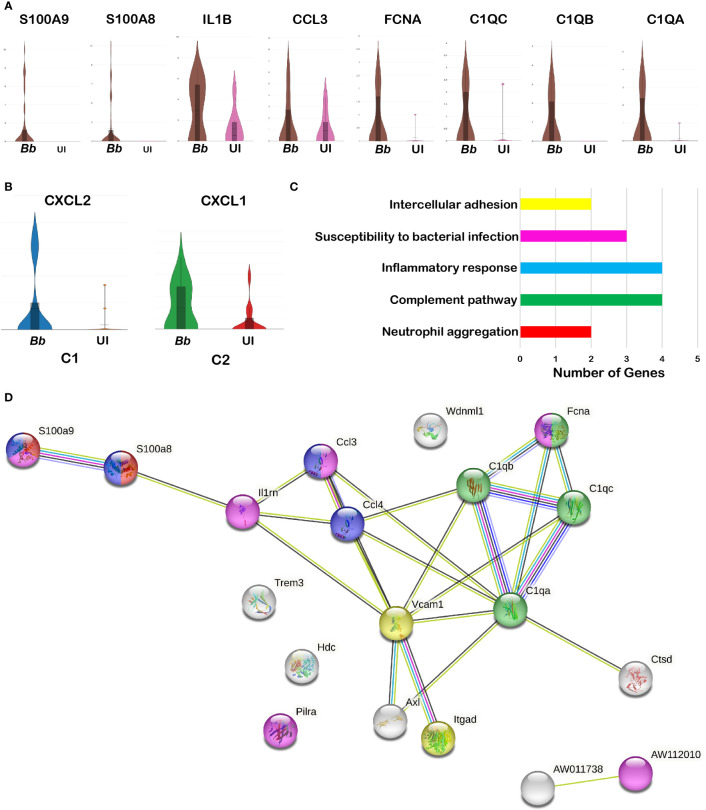
Comparison of the infected and bystander macrophages clusters. Violin plots showing the log2 expression and cellular distribution of the selected DEGs compared between the infected (*Bb*) and bystander (UI) in **(A)** cluster 3 macrophages. **(B)** Cxcl2 in cluster 1 and Cxcl1 in cluster 2. STRING-protein network of upregulated genes identified from the *Bb* infected cluster 3 macrophages, showing the **(C)** functional pathways enriched where each bar indicates the number of identified genes involved in each pathway and **(D)** protein–protein interaction map, where colors in the spheres corresponds to the bar **(C)** representing the functional pathway.

Several significant DEGs were differentially expressed between infected and bystander macrophage in cluster 3 (**Supplementary Table S5B**). Among the top 50 upregulated genes in the total macrophage population in the infected sample, genes encoding complement-related proteins such as Fcna, C1qa, C1qb, and C1qc were significantly upregulated compared to the bystander population (**Figure 7A**). Other significant upregulated genes include S100a8, S100a9, Il1b, and ccl5. Moreover, the genes that are enriched in cluster 3 of neutrophils were also enriched in the macrophage cluster, indicating that these inflammatory macrophages likely be involved in the phagocytosis of cluster 3 neutrophils that are undergoing apoptosis. In addition, clusters 1 and 2 had genes encoding Cxcl2 and Cxcl1, respectively, upregulated in *Bb*-infected cells (**Figure 7B**).

Protein network analysis of the DEGs identified from *Bb*-infected cluster 3 macrophages identified with inflammatory gene markers indicated that the upregulated genes were tightly connected (**Figure 7D**). Moreover, several of these genes were involved in biological processes such as Fc-receptor-mediated stimulatory signaling pathway, regulation of T-cell proliferation, neutrophil chemotaxis/aggregation, myeloid leukocyte activation, inflammatory response, and apoptosis based on protein network analysis (**Figure 7C**).

### Activation of complement genes and cytokines in bone marrow macrophages

**Figure 8:**
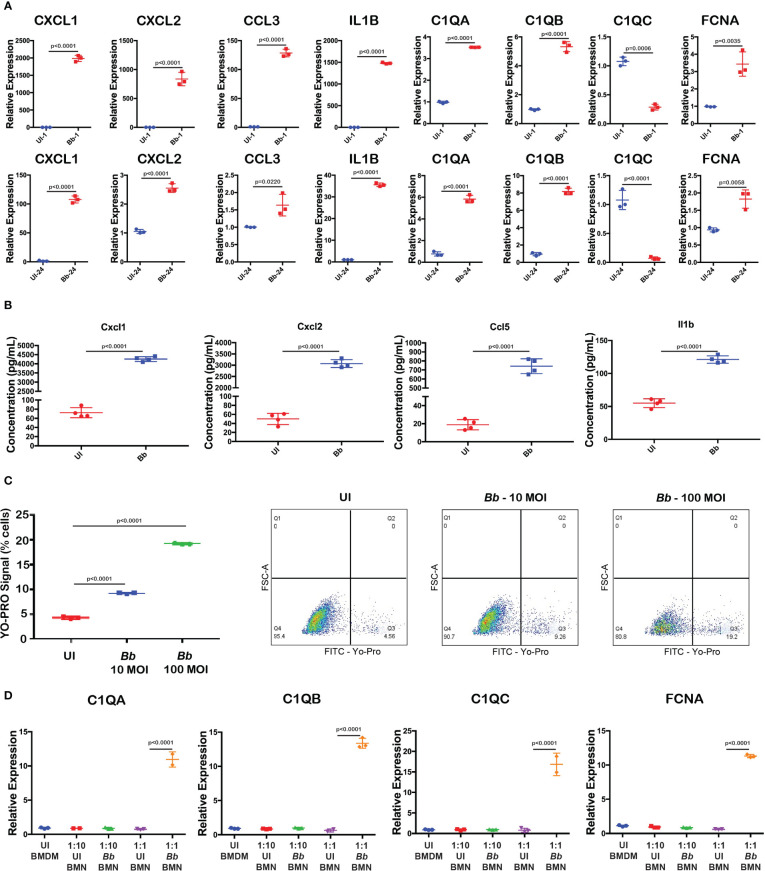
Validation of *Bb*-induced gene modulation in BMNs. **(A)** Gene expression analysis of selected complement and cytokine encoding genes using qRT-PCR analysis comparing uninfected and *Bb*-infected BMDMs at 1 and 24 hpi. **(B)** Levels of cytokines Cxcl1, Cxcl2, Ccl5, and Il1b were determined by ELISA from the culture supernatant of BMDM with or without *Bb* infection. **(C)** Percentage of cells positive for YO-PRO signals in BMNs infected with *Bb* (10 MOI) and *Bb* (100 MOI) compared to the uninfected control. In Dot-Plots, Q3 represents YO-PRO^hi^ cells, and Q4 represents YO-PRO^−^ cells, and the values in each quadrant represent the percentage of cells. **(D)** Gene expression analysis of complement genes in BMDMs co-cultured with *Bb*-infected BMNs at two different ratios of BMN:BMDM—1:10 and 1:1. All qRT-PCR experiments were performed independently twice, and the data displayed are representative of one experiment showing the average values of at least three technical replicates. Data from second experiment are presented in **Supplementary Figures 5**, **6**. Error bar represents the standard deviation between the technical replicates. The p-values in each graph represents the significance of differences between the *Bb*-infected and bystander populations.

scRNA-Seq analysis revealed that genes encoding inflammatory chemokines and complement factors were specifically upregulated in the *Bb*-infected macrophages. To validate the mRNA abundance of select genes, in macrophages during *Bb* infection, qRT-PCR analysis was performed with BMDMs infected with 100 MOI of WT-*Bb*, and RNA was isolated at 1 hpi and 24 hpi. Uninfected BMDM at the corresponding timepoints were maintained as controls. Using the respective cDNA templates, the mRNA abundance of C1qa, C1qb, C1qc, Fcna, Cxcl1, Cxcl2, Ccl3, and Il1b were determined by qRT-PCR using the gene-specific primers, while ß-actin gene was used as normalization control. While C1qa and C1qb were significantly upregulated in *Bb*-infected BMDMs, compared to the uninfected BMDM at 1 hpi and 24 hpi, there was no upregulation of C1qc (**Figure 8A** and **Supplementary Figure S5**). Moreover, lectin complement pathway activation gene, Fcna, is significantly upregulated at 24 hpi than at 1 hpi, suggesting the potential for the activation of lectin pathway. Cytokine genes, Il1b, Cxcl1, Cxcl2, and Ccl3, were significantly upregulated at 1 hpi and 24 hpi in *Bb*-infected BMDMs, although the levels of these four cytokine genes were higher at 1 hpi than at 24 hpi.

To confirm the upregulation of cytokines, Cxcl1, Cxcl2, Ccl5, and Il1b, we performed ELISA and quantified the cytokines levels in the culture supernatant of BMDM infected with *Bb* (100 MOI) for 24 h. Results indicated that the levels of all four cytokines were significantly higher in the *Bb*-infected BMDMs compared to the uninfected BMDMs at 24 hpi (**Figure 8B**).

The protein network analysis indicated that apoptosis-related genes are upregulated in macrophages during *Bb* infection. To further define the role of caspase3-dependent apoptosis in macrophages during *Bb* infection, BMDM was infected with WT-*Bb* (10 and 100 MOI) for 24 h, stained with YP-1, and flow cytometric analysis was performed. As shown in **Figure 8C**, YO-PRO+ cells are significantly higher in *Bb*-infected BMDMs compared to the uninfected BMDMs, indicating levels of apoptotic activity. Compared to 10 MOI infected BMDMs, 100 MOI infected BMDMs exhibited higher YP-1 cells, indicating that *Bb* induces apoptosis in a dose-dependent manner.

### *Bb*-infected neutrophils upregulate complement genes in macrophages

We observed that cluster 3 macrophages expressed both neutrophil-specific genes such as s100a8/s100a9 and Ly6G along with macrophages gene markers including ITGAM and CSF1R in *Bb*-infected splenocytes (**Figure 7A**). Protein–protein interaction analysis showed the activation of neutrophil aggregation pathway in *Bb*-infected cluster 3 (**Figure 7C**). In *Bb*-infected cluster 3, complement genes C1qa, C1qb, C1qc, and Fcna were upregulated compared to bystander cluster 3 macrophages, indicating the possibility of phagocytic macrophages clearing apoptotic neutrophils by complement-dependent efferocytosis process (72). To understand the role of *Bb*-infected neutrophils on the complement activation in macrophages, BMNs were infected with *Bb* at 100 MOI, and at 16 hpi, the infected BMNs were washed with PBS to remove the extracellular spirochetes and co-cultured with BMDM for 2 h, while BMDM co-cultured with uninfected BMNs were maintained as control. qRT-PCR analysis of *Bb*-infected or the uninfected BMNs co-cultured with BMDMs for 2 h showed that C1qa, C1qb, C1qc, and Fcna were significantly upregulated in BMDM that are exposed to higher *Bb*-infected BMNs (1:1—BMN:BMDM) compared to the BMDM exposed to lesser-infected BMNs (1:10—BMN:BMDM) or uninfected BMNs (**Figure 8D** and **Supplementary Figure S6**). The upregulation of C1qc in BMDM exposed to *Bb*-infected BMNs indicates that C1qc likely responds differentially following direct *Bb* infection and/or exposure to *Bb*-infected neutrophils (73).

### Heterogeneity in splenic B cells

**Figure 9:**
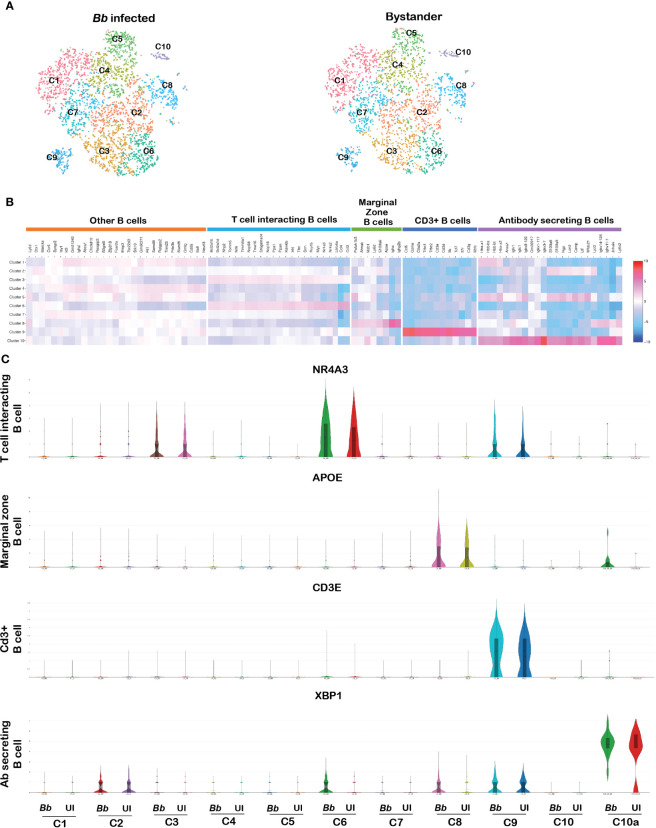
Cell cluster analysis of B cells. Cells filtered for PTPRC+ CD19+ were annotated as B cells. **(A)** tSNE plot of B cells showing 10 clusters marked as C1–C10. **(B)** Heatmap showing the DEGs among the 10 clusters of B cells, and the putative names of each cluster were mentioned over the respective blocks. **(C)** Violin plots showing the log2 expression and cellular distribution of the selected cluster-specific genes.

B-Lymphocyte antigen CD19 is an IgSF surface glycoprotein of 95 kDa that is expressed from the earliest stages of B-cell development (74). Because of the ubiquitous expression of CD19 on the surface of majority of B cells, CD19 was used as the molecular marker to identify B cells among the splenocytes. CD19+ cells contribute the major cell population of total splenocytes (**Supplementary Figure S2B**). The tSNE plot revealed 10 clusters of splenic B cells following reclustering of cells with PTPRC+CD19+ bar codes (**Figure 9A**). Clusters C5, C6, C8, C9, and C10 showed significant DEGs among the 10 B-cell clusters (**Figure 9B**). The list of DEGs upregulated in each B-cell cluster is tabulated in **Supplementary Table S6**.

Cluster 5 expressed genes such as VPREB3 and SIGLECG, which are generally expressed in the precursor B cells (75). Among cluster 6 cells, there was enrichment of NR4A3, NR4A2, LILRB4A, ATF3, RRP1B, CCL3, CCL4, and MYC transcripts. Both NR4A1 and NR4A3 are implicated in restraining B-cell access to T cell help by repressing expression of the T-cell chemokines CCL3 and CCL4. Therefore, this cluster was annotated as T-cell interacting B cells (76). Cluster 8 expressed genes such as MZB1 encoding marginal zone B and B1 cell-specific protein along with other genes such as APOE, S100A6, AHNAK, and PAFAH1B3, which are involved in the activation of T lymphocytes by antigen presentation, termed as marginal zone B cells. Cluster 9 expressed genes such as CD3E, CD3D, CD3G, IL7R, TRBC2, TRAC, and TCF7 that are related to T-cell proliferation and T-cell receptors, termed as CD3+ B cells (77). Finally, in cluster 10, there were two subsets of cells—in one subcluster, there was increased expression of antibody-related genes such as IGKC, IGHM, IGLC1, IGLV1, JCHAIN, FAM214A, and XBP1, reflecting antibody-secreting cells (ASCs) (78). The other subcluster within cluster 10 expressed neutrophil-specific genes, designated as 10a; there were increased levels of XBP gene transcripts (**Figure 9C**). Several genes were expressed at similar levels in clusters C1, C2, C4, and C5 limiting further resolution of these clusters. DEG analysis between infected and bystander populations revealed no significant difference in any of the CD19 clusters presumably due to the short time of interaction between B cells and spirochetes.

### Heterogeneity in splenic T cells

**Figure 10:**
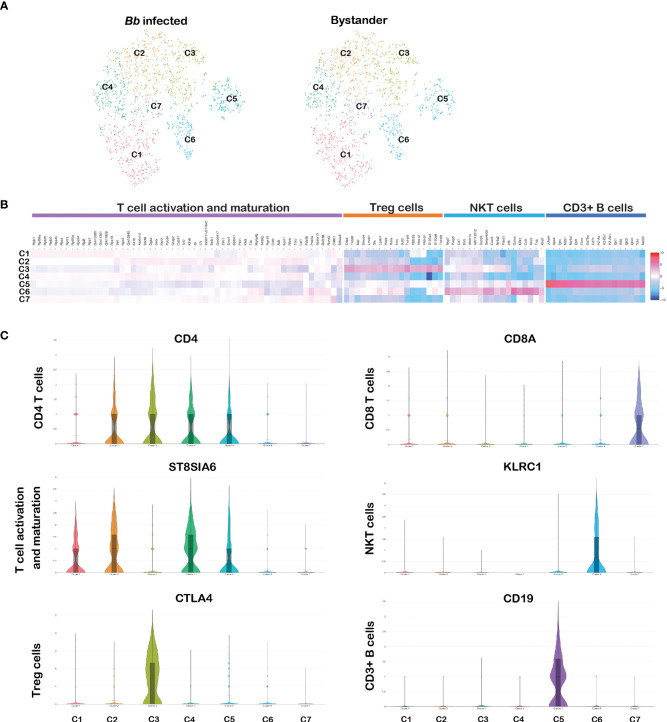
Cell cluster analysis of T cells. Cells filtered for PTPRC+ CD3e+ were annotated as T cells. **(A)** tSNE plot of B cells showing seven clusters marked as C1–C7. **(B)** Heatmap showing the DEGs among the seven clusters of T cells, and the putative names of each cluster were mentioned over the respective blocks. **(C)** Violin plots showing the log2 expression and cellular distribution of CD4 and CD8a genes and other selected cluster-specific genes.

CD3 complex is the T-cell co-receptor that is involved in activating both the cytotoxic T cell and T-helper cells and therefore serves as an ideal marker to distinguish T cells from other populations (79). In the original tSNE plot, clusters 3, 9, 10, 11, and 16 expressed CD3e gene as shown in **Figures 1A–C**. To differentiate cells within the T-cell clusters, we filtered PTPRC+CD3E+ cells from the total splenocytes and further tSNE plots/analysis revealed seven clusters (**Figure 10A**). Significant DEGs were observed between the seven clusters (**Figure 10B**). The list of DEGs upregulated in each T-cell cluster is tabulated in **Supplementary Table S7**.

Among them, clusters 1–5 expressed CD4 gene representing the CD4 subset of T-helper cells. As shown in **Figure 10C**, cluster 3 expressed CD4^+^ regulatory T (Treg) cell-specific genes encoding cytotoxic molecules such as TNFRSF4, TNFRSF9, CTLA4, S100A4, and IKZF2, and select genes expressed in Treg cells such as FOXP3, IL17A, and IL17F (80). Cluster 1, 2, and 4 expressed similar transcriptional patterns including significantly higher expression of ST8SIA6, TDRP, and LEF1, which is similar to the expression patterns in mature T cells (81). However, no significant DEGs were observed between these three clusters. Cluster 5 expressed CD19, which is similar to the cluster 9 in the CD3+ B-cell population. CD3^+^CD19^+^ lymphocytes subsets have been identified in disease conditions such as HIV/*Mycobacterium* co-infections (77). Cluster 7 expressed CD8a and CD8b1 representing the cytotoxic CD8 subset of T cells (**Figure 10C**). Cluster 6 was enriched with KLRC2 gene family, located within the NK complex genes, and based on this transcriptome profile, we designated cluster 6 as NKT cells (82). No significant DEGs were observed between infected and bystander CD3e+ cells. Significant changes in gene expression were not anticipated in T cells during bacterial infection as early as 45 min.

In summary, scRNA-Seq analysis of splenocytes exposed to *Bb* for 45 min revealed a large array of genes that are induced, although the clusters of splenocytes are relatively similar between infected and bystander cells. Most notably, the differences in the DEGs induced in splenocytes infected with *Bb* provide a rich platform of genes and associated cell populations whose levels could influence the course of the immune response against *Bb* in a reservoir host. Although these studies are based on *Bb* infection under *ex vivo* conditions, it enabled analysis of cells that were infected compared to bystander cells in sufficient numbers for an unbiased analysis using flow-based sorting and scRNA-Seq methodology in conjunction with validation of RNA, protein, and cytometric analysis using defined populations of cells.

## Discussion

In this study, we exploited the ability of GFP+ *Bb* to “label” sufficient number of murine splenocytes (10,000 cells) following *ex vivo* infection and sorted GFP+ cells from GFP− bystander cells using flow cytometry to determine the effects of *Bb* at a single-cell level (**Figure 1A**). The incubation of GFP+ *Bb* with the splenocytes at 1:1 ratio for 45 min resulted in 23% of GFP+ splenocytes (**Figure 1B**), which was similar to intracellular localization of *Bb* in human endothelial cells reported previously (15). The GFP+ splenocytes reflect either interactions of intact *Bb* or processed following intracellular uptake resulting in splenocytes labeled with GFP to be sorted as “infected.” It is well established that *Bb* is an extracellular pathogen primarily surviving in the extracellular matrices of the host with no replication of *Bb* within host cells in large numbers as would be observed for intracellular or facultatively intracellular bacterial pathogens (83, 84). The dissemination of spirochetes from the site of deposition on the skin following a tick bite involves a multistep dynamic process of tethering, dragging, adhesion, and extravasation in conjunction with the motility of the pathogen (85) Prior studies have demonstrated the attachment of *Bb* to host cells surfaces and uptake and processing of *Bb* within various intracellular compartments (11, 22, 86–88). Using scRNA-Seq, it was possible to dissect the early cellular and transcriptome landscape of splenic immune cell populations of C3H/HeN mice that interact with *Bb* to establish an atlas of cells that drive the subsequent innate and adaptive immune responses of the host following active infection.

The tSNE plots defined 28 different clusters, comprising of B cells, T cells, neutrophils, macrophages, NK cells, and erythrocytes that interacted with GFP-expressing spirochetes identified by two independent scRNA-Seq analysis (**Figures 2A, B**). There were significant differences in a large number of DEGs between infected and bystander splenocytes from experiment 1 compared to experiment 2, and therefore, we focused on validating a majority of DEGs that were significantly different between these populations identified in experiment 1 while providing the scRNA-Seq data sets for both experiments. The percentage of each cell population was consistent with the previous findings on murine splenocytes from different strains of mice (49). Furthermore, the clusters identified using the scRNA-Seq data included majority of splenocytes, and the identity of these clusters was validated using cell-specific markers (**Figure 2C**). Notably, it was feasible to classify PTPRC+ cells based on the expression of unique markers as shown in **Figure 2C** into different cell populations. A comparison of early transcriptomic modulations of each cell population between GFP+ (infected) and GFP− (bystander) splenocytes, exhibited similar cellular landscape with no population specific for *Bb* infection (**Supplementary Figure 2A**). This indicated that *Bb* could infect several subsets of splenic cell types as early as 45 min albeit under *ex vivo* conditions. Previous studies showed the ability of *Bb* to infect immune, phagocytic, non-immune, and non-phagocytic cells (89). Although there is no exclusive cell population identified among *Bb*-infected splenocytes, the count of *Bb*-infected neutrophils and macrophages was significantly higher, suggesting the importance of myeloid cells during early stages of innate immune response following *Bb* infection (**Supplementary Figures 2B**, **3**). As expected, neutrophils are the first responders to be recruited with significant microbicidal activity followed by an influx of monocytes/macrophages to resolve the inflammation at the site of *Bb* infection (33). While *Bb* could interact with different subpopulations of immune and non-immune cells, we could not uncover the difference in the mechanism of interaction of *Bb* with each cell type. However, Tlr2, one of the critical pathogen recognition receptors (PRRs) of the host capable of interacting with pathogen-associated molecular patterns (PAMPs) of *Bb*, was expressed only in the myeloid cell populations of infected splenocytes as depicted in cluster 22 (**Figures 2E, F**; **Supplementary Table S3**).

### Cellular heterogeneity of neutrophils

A comparison of infected and bystander populations helped to classify various clusters of neutrophils into inflammatory (C1), antigen presenting (C2), apoptotic (C3), and progenitor neutrophils (C4) based on their transcriptome signatures (**Figures 3A, B**) providing additional insights on the role of neutrophil populations in the host response against Lyme disease agent. While increased infiltration of polymorphonuclear leukocytes in the synovial fluid occurs during infectious stages of Lyme arthritis in humans, a higher percentage of mononuclear leukocytes and lymphocytes is observed during post-infectious stages reflecting cellular profile changes that contribute to tissue pathology (90). Moreover, the host inflammatory response in the joints due to dissemination of Lyme spirochetes in the murine model is characterized by the infiltration of mainly neutrophils and macrophages (91). Neutrophils are short-lived inflammatory cells programmed to undergo apoptosis, perhaps to limit their proinflammatory response (33). However, prolonged proinflammatory response via activation of neutrophils and other cellular mediators of immune response induced by either intact spirochetes or its breakdown products have been suggested to sustain pathogenesis observed in Lyme arthritis (5). The functional relevance of individual clusters of neutrophils recognized in this study in modulating the host response to *Bb* infection can be exploited for diagnosis or prognosis of Lyme disease (92).

### *Bb* infection activates caspase 3 expression in neutrophils

Among the four clusters of the neutrophils (**Figure 4A**), C3 had DEGs that could be readily connected to functional pathways such as those involved in apoptosis, NFkB/TNF/IL17/TLR signaling and those that contribute to rheumatoid arthritis (**Figures 4C, D**). We further validated the levels of DEGs involved in apoptotic mechanisms such as Casp3 and pro-inflammatory cytokines released by neutrophils such as Cxcl1, Cxcl2, Ccl5, and Il1b, which have been shown to play a role in Lyme arthritis (93). Apoptosis, or programmed cell death, is an important mechanism contributing to the resolution of inflammation (94, 95). Prior studies demonstrated that clearance of apoptotic cells is important for the resolution of inflammation during experimental Lyme arthritis (33). It was also shown that increasing the recruitment of neutrophils to the site of infection dramatically attenuates *Bb* infectivity (96). Our data indicated that *Bb*-infected cluster 3 neutrophils had elevated expression of HCAR2, CXCL2, DUSP1 and IL1B genes, compared to bystander splenocytes (**Figure 4B**). We further identified the upregulation of select DEGs that are involved in initiating apoptotic process indicating that neutrophils undergo apoptosis during *Bb* infection based on the protein–protein interaction pathways predicted (**Figure 4D**). However, when we analyzed the relative expression of effector caspases by qRT-PCR (**Figure 5A**), Casp3 was upregulated at 16 hpi and 24 hpi, suggesting a more prominent role for Casp3 during *Bb* infection in BMNs (27). Furthermore, flow cytometry analysis using YO-PRO-1 iodide stain showed that *Bb*-infected BMNs undergo Casp3-dependent apoptosis in a dose-dependent manner (**Figures 5B, C**). Moreover, 10 µg/mL of purified borrelial lipoproteins (*Bb*Lp) also induced Casp3-dependent apoptosis in BMNs (**Figure 5D**). These results showed that BMNs exposed to either intact *Bb* or *Bb*Lp undergo caspase 3-dependent apoptosis. In addition, flow cytometric analysis indicated that only 10%–20% of BMNs were positive for YP-1, suggesting that *Bb* infection might induce apoptosis only in a sub-cluster of neutrophils (**Figures 5B, C**). scRNA-Seq analysis showed that cluster 3 neutrophils contributed approximately 10%–25% of total neutrophils. which are potentially involved in the activation of these pathways in splenocytes, suggesting that cluster 3 could play a modulatory role influencing the survival of spirochetes in mammalian hosts.

The role of DUSP1 in contributing to the host response against *Bb* is yet to be appreciated. Flow cytometry analysis showed that inhibition of DUSP1 has a significant impact on the BMN granularity and does not have any impact on *Bb-*induced caspase activation. *Bb* infection in BMNs induced a specific FSC-SSC-hi population, representing high granular cell population (**Figure 5E**), while BCI treatment (DUSP1/6 inhibitor) of *Bb*-infected BMNs reduced the FSC-SSC-hi population (**Figure 5F**). Moreover, BCI treatment significantly reduced the FSC-hi population in uninfected BMNs (**Figure 5G**). These results suggested that DUSP1 plays an important role in maintaining the granularity in neutrophils during *Bb* infection.

Interestingly, BCI inhibitor treatment significantly increased YP-1-hi population in BMNs independent of *Bb* infection, suggesting that inhibition of DUSP1 increases Casp3 activation in neutrophils (**Figures 5G, H**). However, in *Bb*-infected neutrophils, BCI treatment did not change the YP-1-lo population, suggesting that *Bb*-induced Casp3 activation was unaffected by BCI treatment. Robitaille et al. demonstrated that DUSP1 regulates apoptosis during virus infection, and therefore, the role of DUSP1 in *Bb* colonization in mammalian host warrants additional studies (97).

### Activation of Cxcl1/Cxcl2/Il1b cascade in neutrophils by *Bb*

*Bb* infection induced the expression of cytokines Cxcl1 and Cxcl2 along with other cytokines such as Il1b and Ccl5 as early as 1 hpi in neutrophils (**Figure 5I**). Previous studies have shown that Cxcl1/Cxcl2/Il1b axis in neutrophils plays a major role in inflammatory response during bacterial infections (98). Boro et al. also demonstrated that Cxcl1 and Cxcl2 regulate NLRP3 inflammasome activation, a key pathway involved in the inflammatory response (99). Although the role of Cxcl1 in recruiting neutrophils during *Bb* infection has been demonstrated, the increased expression of Cxcl1/Cxcl2/Il1b axis in neutrophils during *Bb* infection is a novel observation from this scRNA-Seq analysis (100). In addition, our results also indicated that *Bb* infection upregulated Ccl5 in neutrophils (**Figure 5I**). Moreover, a novel finding from this study is the identification of subset-specific expression of Cxcl1 (cluster 2), Cxcl2 (cluster 1), and Il1b/Ccl5 (cluster 3) in neutrophils during *Bb* infection, indicating the independent roles of these cytokines during *Bb* infection. Murine Lyme arthritis is strongly dependent on IL-1 production, and *Bb* induces TLR2/MyD88-dependent inflammasome-mediated caspase-1 activation in BMDM (101, 102). However, caspase 1 deficiency in mice had no effect on *Bb*-induced humoral immunity, T-cell responses, or the abilities of macrophages to ingest and degrade spirochetes (103).

Analysis of scRNA-Seq data and validation by qRT-PCR showed that DUSP1 is upregulated at 1 h, 16 h, and 24 h post-infection reflecting its role in modulating the pro-inflammatory cytokine expression in BMNs during *Bb* infection (**Figure 5I**). BCI (DUSP1/6 inhibitor) treatment elevated the Cxcl1/2 cytokines, indicating that DUSP1 negatively regulates the Cxcl1/2 pro-inflammatory signaling axis in neutrophils during *Bb* infection (**Figure 5J**). In contrast, BCI treatment completely reduced the Ccl5 expression in *Bb* infected neutrophils, indicating that Ccl5 expression during *Bb* infection is driven by the upstream DUSP1-mediated signaling cascade (**Figure 5J**). Interestingly, BCI treatment did not affect the Il1b expression in *Bb*-infected neutrophils, presumably due to Il1b expression occurring independent of DUSP1 pathway. Nengyin et al. (103) demonstrated that *Bb*-infection-induced expression of Il1b is independent of NLRP3, NOD1, NOD2, and RICK correlating with our findings. DUSP1 silencing increased Il1b-induced MAPK phosphorylation and ZFP36 expression at 2 h and profoundly repressed TNF mRNA at 6 h in A549 cells (104, 105). However, there is limited information about the regulation of Il1b in murine bone-marrow-derived neutrophils. Studies have confirmed the essential functions of DUSP1 in controlling the anti-microbial innate immune responses. Utilizing DUSP1(−/−) mice, Rodriguez and others demonstrated that DUSP1 is an essential negative regulator of TLR-triggered innate immune activation in chlamydial infection promoting chlamydial growth through direct effects on infected cells (106). Overall, the above findings suggest the possibility of Cxcl1, Cxcl2, Il1b, and Ccl5 to be functionally independent of each other driven by different pathways during *Bb* infection in neutrophils.

### Cellular heterogeneity of macrophage population among splenocytes

Among the PTPRC+ splenocytes, CSF1R was used to classify 5 macrophage clusters (**Figure 6A**). All clusters identified had similar levels of CSF1R and ITGAM (CD11b) reflecting expression of macrophage-specific markers (**Figure 6B**). Moreover, cluster 3 classified as inflammatory macrophages expressed DEGs specific to complement pathway components (C1qa, C1qb, C1qc, and Fcna), macrophage inflammatory, and activation proteins, and neutrophils attractants such as Cxcl1 and Cxcl2 (**Figure 6C**). Interestingly, exosomes-specific marker CD63 was upregulated in cluster 3, suggesting that these clusters are involved in exosomes formation. Macrophages-derived exosomes are involved in controlling inflammation by releasing several damage-associated molecular patterns (107). Cluster 3 also had several DEGs that were significantly upregulated in infected macrophages compared to bystander macrophages (**Figure 6A**). Overall, the identification of macrophage clusters among splenocytes with higher expression of select DEGs in infected population reflects the role of macrophages in initial stages of *Bb* infection.

### Pathogen-induced responses in inflammatory macrophages

As shown in **Figures 7A, D**, inflammatory macrophages (cluster 3) exhibited a highly interconnected set of unique DEGs encoding for neutrophil granule proteins such as s100a8 and s100a9 that are upregulated in neutrophils in response to infection. These same factors are also upregulated in apoptotic neutrophils that have phagocytosed bacterial pathogens (53). The association of cluster 3 neutrophil markers with cluster 3 macrophages is suggestive of an intriguing possibility of macrophages controlling inflammatory responses by efferocytosis of apoptotic neutrophils (**Figures 7A, D**). In addition, genes encoding intercellular adhesion molecules such as VCAM1, and its receptor ITGAD, are upregulated in inflammatory macrophages (**Figures 7C, D**) (108). In addition, genes encoding trigger receptor expressed on myeloid cells 3 (TREM3) and cytokines such as Ccl3, Ccl4, and Il1rn [IL1 receptor antagonist that inhibits the activities of interleukin 1 alpha (IL1A) and interleukin 1 beta (IL1B) inhibiting IL-1-related inflammatory responses in macrophages] are upregulated in *Bb*-infected cluster 3 macrophages. Recently, Liu et al. identified IL1RN+/TREM1+ macrophage subpopulation via blockade of TREM1 serving as a therapeutic tool for treatment for thoracic aortic aneurysm and dissection (109). Another novel finding is the increased levels of long non-coding RNAs AW011738 and AW112010 in the infected cluster 3 macrophages (**Figure 7D**). The lncRNA AW112010 has been implicated in the functions of macrophage in aging-related processes (110). These aforementioned DEGs provide avenues for exploring their contributions in regulating macrophage responses in inflammatory processes induced by *Bb* infection.

Prior studies demonstrated that the activation of Cxcl1/Cxcl2 in murine macrophages is a key event during *Bb* infection (111). The increased levels of these chemokines in infected macrophage clusters 1 and 2 also add to the relevance of other macrophage clusters in the inflammatory responses against infection (**Figure 7B**). Several DEGs specific to cytokines Cxcl1, Cxcl2, Ccl3, and Il1b were upregulated in bone-marrow-derived macrophages at both 1 and 24 h post-infection with *Bb* validating the mRNA abundance observed in scRNA-Seq analysis of infected splenocytes (**Figure 8A**). The mRNA abundance of select DEGs were also validated at protein level using ELISA-based assays, and levels of Cxcl1, Cxcl2, Ccl5, and Il1b were all upregulated in infected BMDMs (**Figure 8B**). Chung et al. demonstrated that *Bb*-elicited IL10 suppresses the Cxcl1/Cxcl2 activation in macrophages leading to a dampening of the inflammatory responses (112). Murine Lyme arthritis is strongly dependent on Il1b; production, and *Bb* induces TLR2/MyD88-dependent inflammasome-mediated caspase-1 activation in BMDM (101, 102). Together, the expression pattern of Cxcl1/Cxcl2/Il1b during *Bb* infection in BMDM was consistent with the earlier studies, indicating the utility of this scRNA-Seq analysis for mining the transcriptome responses that can be exploited to identify different cell types in a mixed population of cells responding to *Bb* infection.

### *Bb*-induced apoptosis in macrophages

Based on the DEGs related to apoptosis pathway in cluster 3 macrophages, we hypothesized that macrophages might also undergo apoptosis during *Bb* infection like cluster 3 neutrophils. We utilized YP-1 to determine the effector caspases activation in BMDM upon *Bb* infection, and we observed that *Bb* induce Casp3 activation in BMDM in a dose-dependent manner, indicating that 10%–20% of BMDM undergo Casp3-dependent apoptosis (**Figure 8C**). Infection of C3H/HeN mice with *Bb* induces an increase in apoptotic macrophages and apoptotic neutrophils, which correlates with arthritis inflammation and clearance of these apoptotic macrophages and neutrophils correlates with the resolution of arthritis (33). These findings suggested that cluster 3 macrophages undergoing apoptosis might be important for the resolution of inflammation.

### Activation of complement components in inflammatory macrophages by *Bb*

Another set of upregulated genes in cluster 3 macrophages is the one that encodes complement protein C1qa, C1qb, C1qc, and Fcna (**Figure 7A**). qRT-PCR results indicated that C1qa and C1qb genes are highly expressed in *Bb*-infected BMDM, while C1qc did not show significant difference in the expression both at 1 hpi and 24 hpi. While C1qc was not upregulated in BMDMs infected with *Bb*, it was found to be upregulated when BMDMs were exposed to *Bb*-infected BMNs, suggesting the functional relevance of C1qc during the process of efferocytosis rather than during direct phagocytosis of *Bb* by macrophages (**Figures 8A, D**). These data suggest the role of C1q proteins in controlling the *Bb* infection by macrophages via multiple cellular processes during bacterial infections.

Prior studies have demonstrated that complement protein C1q directs macrophage polarization and limits inflammasome activity during the uptake of apoptotic cells (113). In addition, C1q-mediated classical complement pathway was shown to be critical to control *Bb* during experimental infection in mice (114). These studies collectively suggest the differential roles of C1q components during *Bb* infection. scRNA-Seq data showed that Ficolin A (Fcna), a lectin complement component, was upregulated in *Bb*-infected macrophages. Fcna recognizes N-acetyl compounds such as N-acetylglucosamine, components of bacterial and fungal cell walls (115). Binding of the ficolin-MBL-associated serine proteases (MASPs) complex to carbohydrates present on the surface of bacteria initiates complement activation via the lectin pathway. Prior studies showed that increased levels of Ficolin-1 at the mRNA and protein levels exerted a protective role against rheumatic fever via bacterial elimination (116). Xu et al. reported that LPS stimulation induced increased expression of FcnA in alveolar macrophages (117). On the other hand, Fcna expression in macrophages also predisposed patients with rheumatic heart disease symptoms for chronic inflammation and tissue injury emphasizing Fcna in bacterial pathogenesis (116). Complement component C1q, in the absence of other complement components, can trigger a rapid enhancement of phagocytosis of apoptotic cells, independent of its ability to activate the complement pathway (118). This indicates that *Bb* infection in splenocytes activated both classical and lectin-mediated complement pathways in macrophages, which may lead to apoptosis.

In addition, we demonstrated that the C1q encoding genes, C1qa, C1qb, and C1qc, were significantly upregulated in BMDMs that are exposed to *Bb*-infected BMNs. This suggested that complement pathway is activated in macrophages when they are exposed to *Bb*-infected apoptotic neutrophils. Zhi et al. (119) demonstrated that complement component, C1q, and the classical complement pathway play important roles in controlling borrelial infection via antibody and complement-dependent killing and altering both antibody maturation processes and the T-cell response following exposure to infectious *Bb*. Our observations suggest the likelihood that cluster 3 macrophages might play a key role in clearing the *Bb*-infected neutrophils via complement activation-mediated phagocytosis. Furthermore, our experimental evidence indicated that complement components are significantly upregulated in BMDM exposed to BMNs infected with *Bb* compared to BMDMs exposed to uninfected BMNs (**Figure 8D**). Protein network analysis indicated that genes involved in the regulation of T cells were upregulated in cluster 3 macrophages, suggesting their putative role in T-cell activation during *Bb* infection via intercellular adhesion molecules present at the mRNA level in infected cluster 3 macrophages (**Figures 7C, D**). Together, these findings indicate that the cluster 3 macrophages play a key role in clearing the *Bb*-infected neutrophils via complement activation-mediated phagocytosis and contribute to other innate/adaptive immune processes.

*Bb*-infected macrophages shared a transcriptome profile that correlated with that observed in experimental models of atherosclerosis. Inflammatory macrophage cluster identified from an atherosclerosis mouse model showed high-level expression of various genes previously assigned to a pro-atherogenic role (e.g., Ccl3, Il1b, Il1a, Nlrp3, Cebpb, Egr1, and Phlda1) (120). Interestingly, several of the above genes were upregulated in *Bb*-infected macrophages, suggesting a role for inflammatory macrophages in Lyme carditis. A recent study demonstrated that *Bb* infection induces long-term memory-like responses in macrophages with tissue-wide consequences in the heart (121). Together, these findings implicate the relevance of inflammatory macrophages in multiple Lyme complications such as arthritis and carditis; therefore, identifying and targeting the subsets of macrophages might provide avenues for the treatment of Lyme carditis.

### Cellular heterogeneity in B- and T-cell populations among splenocytes

Several clusters of B (CD19+) and T (CD3e+) cells were observed demonstrating the heterogeneity of B cells and T cells in the splenocytes of C3H/HeN mice. Of the splenocytes, 64% were CD19+ cells, indicating that the B cells are the major cellular components of splenocytes. Cell cluster analysis of CD19+ cells revealed 10 different clusters (**Figure 9A**), of which five clusters expressed unique markers, while in the other five clusters, several genes were commonly expressed (**Figure 9B**). Based on the DEGs, CD19+ cells were functionally clustered as antibody-secreting B cells (C2, C8, and C9), CD3+ B cells (C9), marginal zone B cells (C8), and T-cell interacting B cells (C3, C6, and C9). Other B cells clusters did not exhibit any significant DEGs that facilitated their identity. A subset of antibody-secreting B cell cluster, termed as cluster 10a, expressed neutrophils like transcriptome profile and was classified as antigen presenting (AP) neutrophil cluster. This suggested the possibility of B cells interacting with neutrophils (**Figure 9C**). Puga et al. demonstrated that B-helper neutrophils stimulate immunoglobulin diversification and production in the marginal zone of the spleen (122). Our findings indicate the likelihood that a subset of splenic neutrophils might be involved in the antigen-presenting functions interacting with the B cells. Notably, we identified that immunoglobulin encoding genes such as Iglc2, Igkv8-27, Ighv1-82, and Ighg2b were specifically upregulated in *Bb*-infected antigen-presenting neutrophil cluster. The functional significance of the role of these genes in controlling *Bb* infection is unknown. However, DEGs expressed between infected and bystander cells (**Figure 9C**) had no significant difference, suggesting a lack of effect of *Bb* on splenic B cells during early *Bb* infection employed in this study.

CD3e+ T cells are the second major cellular components of splenocytes, comprising seven sub-clusters (**Figure 10A**) with a significant difference in DEGs among the three clusters, termed Treg cells (cluster 4), NK cells (cluster 6), and CD3+ B cells (cluster 5) (**Figure 10B**), as these three clusters are significantly different from classical lymphoid T cells. Cluster 3 expressed Tnfrsf4, Tnfrsf9, Ctla4, s100a4, and Ikzf2 at higher levels compared to the other six clusters (**Figure 10B**). Several of these genes such as Tnfrsf4, Tnfrsf9, Ctla4, s100a4, and Ikzf2 are expressed in Treg cells (123). Therefore, it is possible to categorize cluster 3 T cells as Treg cells. Cluster 5 with significant DEGs is CD3+ B cells cluster (**Figure 10C**). As observed in B-cell populations, a cluster expressing both CD3 and CD19 were classified as CD3+ B cells. While cluster 5 could reflect interaction of B and T cells, it is also possible that both markers are expressed in a single cell. Finally, cluster 6 encompassed NKT cells expressing killer lectin-like receptors (KLR) genes. It has been shown that the heterogeneous but conserved natural killer receptor (KLR) gene complexes are present in four major orders of mammals, highlighting the roles of these genes in NKT cell population (124). Among the other four clusters, we identified that cluster 7 exclusively expressed CD8a gene, while the other three clusters (1, 2, 4) expressed CD4+ cells. Most of the genes that are expressed in these clusters such as ST8SIA6, LEF, PDCD1, and CD247 (**Figure 10B**) are involved in the T-cell activation and maturation (125). Although these subsets of T cells were identified as separate clusters, there are no significant DEGs identified between these four clusters. As expected, due to the short duration of interaction between GFP+ *Bb* and splenocytes, we were unable to identify significant transcriptome changes in T cells from infected and bystander population of splenocytes. However, we expect that these clusters might exhibit differential activation during later stages of *Bb* infection, and further studies are required to understand the roles of these clusters under *in vivo* conditions.

Interestingly, we identified a unique cluster expressing both CD3e and CD19 genes (**Figure 1C**), although no significant DEGs were observed between infected and bystander populations in these clusters. Qing et al. reported that CD3+CD19+ cell counts were observed to be markedly reduced in patients with HIV/*Mycobacterium* co-infection compared to healthy controls (77). They observed that the inhibitory molecules PD1 and T-cell immunoreceptor with Ig and ITIM domains (TIGIT) were highly expressed in the CD3+CD19+ cells in the HIV/*Mycobacterium* co-infection group, indicating a phenotype involving greater suppression of the adaptive immune response. However, further studies are required to understand the functional relevance of this population during *Bb* infection in mice.

## Key findings and conclusions

In this study, we identified and compared the transcriptomic profiles of *Bb*-infected and bystander splenic immune cells at single-cell resolution using GFP+ *Bb*. Several novel splenic immune cell clusters were detected during *Bb* infection as early as 45 min of infection. Our findings indicate that select cellular responses such as apoptosis, complement, and inflammatory pathways are specifically localized to the infected cells providing a rich array of biomarkers/cell clusters that can be targeted to reduce pathological manifestation of Lyme disease in humans. Furthermore, this study provides novel information on the activation of pathogenesis-related signaling pathways in *Bb* infected neutrophils and macrophages cell clusters at early stages of *Bb* infection. Further studies on the identified clusters of innate and adaptive immune cells using knockout mice models will increase the contributions of these clusters during *Bb* infection. These findings provide opportunities to explore the possibilities of targeting novel myeloid sub-populations to limit spirochete survival in mammalian hosts.

## Data availability statement

The datasets presented in this study can be found in Gene Expression Omnibus (GEO) online repository under the accession number GSE243126 containing four samples with accession numbers GSM7779603, GSM7779604, GSM7779605 and GSM7779606.

## Ethics statement

The animal study was approved by Institutional Animal Care Program (IACUC), University of Texas at San Antonio. The study was conducted in accordance with the local legislation and institutional requirements.

## Author contributions

VK: Conceptualization, Formal Analysis, Investigation, Writing – original draft, Data curation, Methodology, Validation, Visualization. TI: Investigation, Writing – original draft. NK: Investigation, Writing – original draft. GZ: Writing – review & editing. BH: Writing – review & editing, Formal Analysis, Methodology. JS: Conceptualization, Funding acquisition, Investigation, Resources, Writing – original draft, Writing – review & editing, Methodology, Project administration, Supervision, Validation.
