# Supplementary material for: Cellular and transcriptome signatures unveiled by single-cell RNA-Seq following ex vivo infection of murine splenocytes with Borrelia burgdorferi
Source: Front Immunol. 2023 Dec 8;14:1296580. doi: 10.3389/fimmu.2023.1296580 (PMC10749944; doi:10.3389/fimmu.2023.1296580)
Supplement: Supplementary file 1 [file DataSheet_1.pdf]

Supplementary Figure 1

10xgenomics – Data analysis workflow

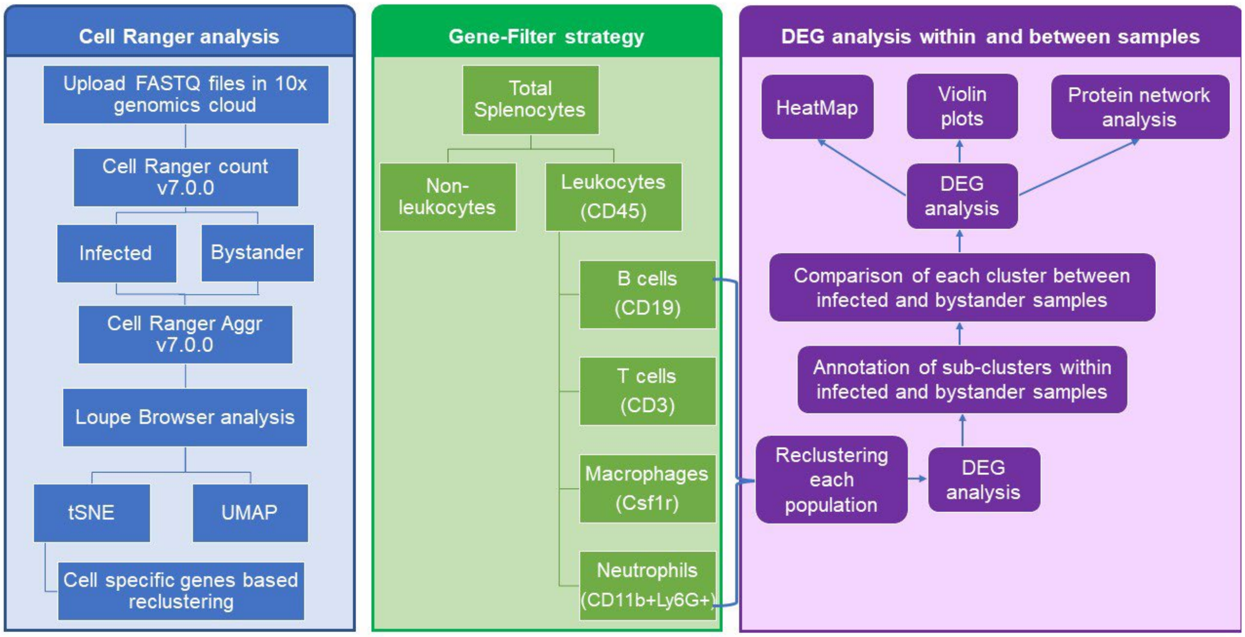

Supplementary Figure 2: Comparison of GFP+ splenocytes and GFP- splenocytes. a) tSNE plots of *Bb* infected (top) and bystander (bottom) splenocytes, b) cell count comparison of *Bb* infected (blue) and bystander (red) barcodes from two independent experiments. Blue bars (Bb infected) and red bars (Bystander) represent the average value of two independent sequencing experiments and the error bar represents the standard deviation between the two experiments. * represents significant differences between *Bb* infect ed and bystander cell populations (p<0.001).

a) Bb vs UI

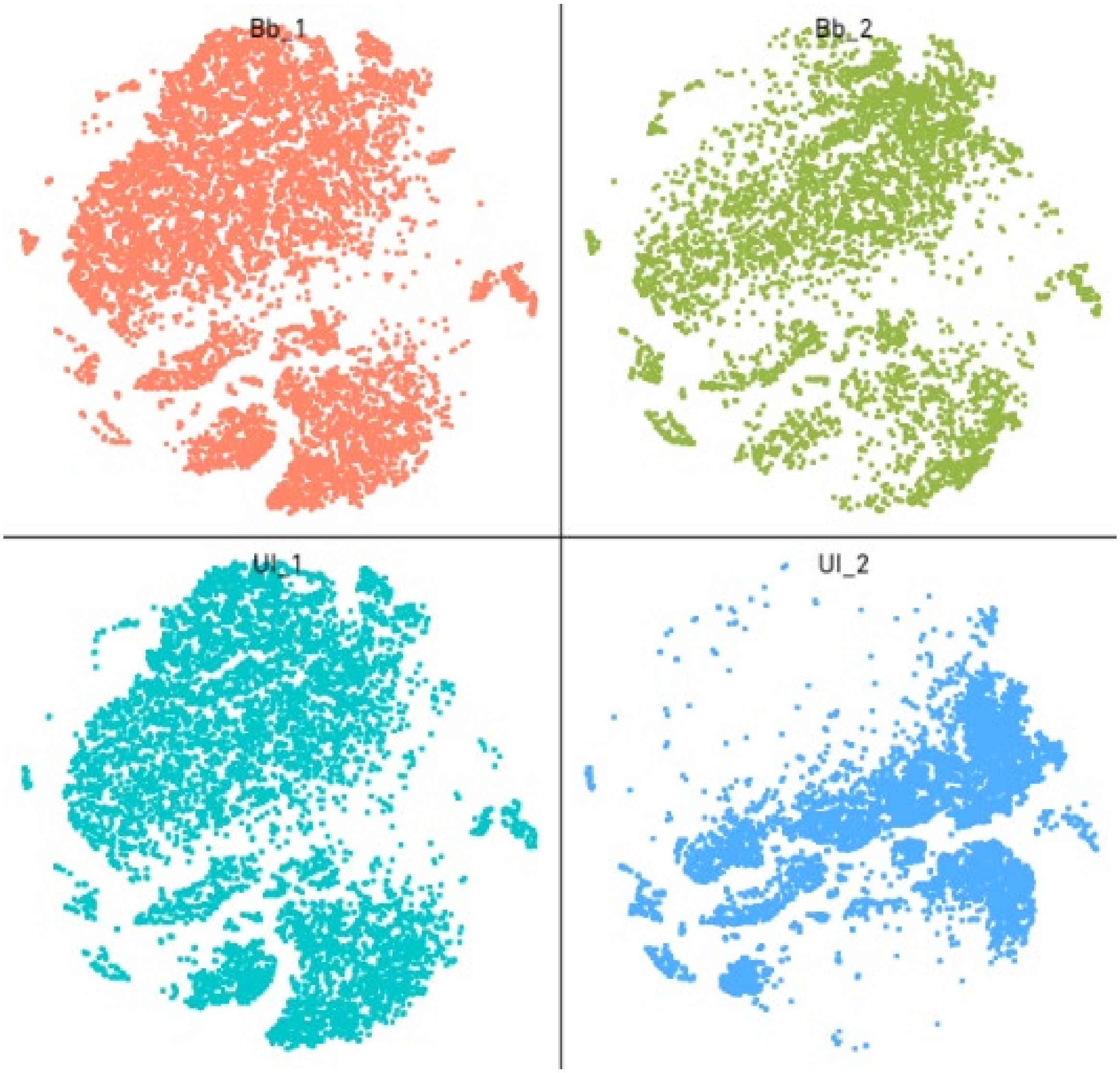

b) Cell count - Bb vs UI

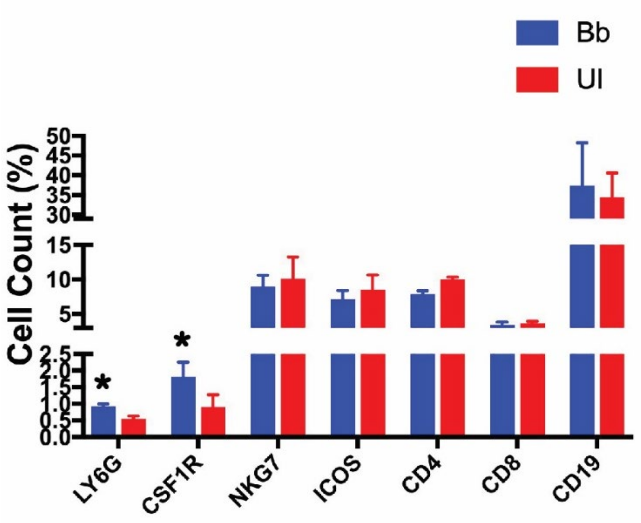

Supplementary Figure 3: To determine the difference in neutrophils and macrophages counts between GFP+ and GFP- splenocytes, splenocytes were infected with GFP-tagged Borrelia for 45 minutes, stained with anti-CD45-PE, anti-CD11b-PE-cy7, anti-Ly6G-APC-Cy7 and anti-F4/80-APC and flow cytometry was performed. A) Gating strategy b) In *Bb* infected splenocytes, GFP+CD11b+F4/80+ macrophages population represented 40.89±3.16% of total GFP+CD45+ leukocytes, while in bystander splenocytes, GFP-CD11b+ F4/80+ macrophages population represented 2.53±0.12% of total GFP-CD45+ leukocytes. Similarly, in *Bb* infected splenocytes, GFP+CD11b+Ly6G+ neutrophils population represented 51.74±4.18% of total GFP+CD45+ leukocytes, while in bystander splenocytes, GFP-CD11b+Ly6G+ neutrophils population represented 3.8±0.33% of total GFP-CD45+ leukocytes.

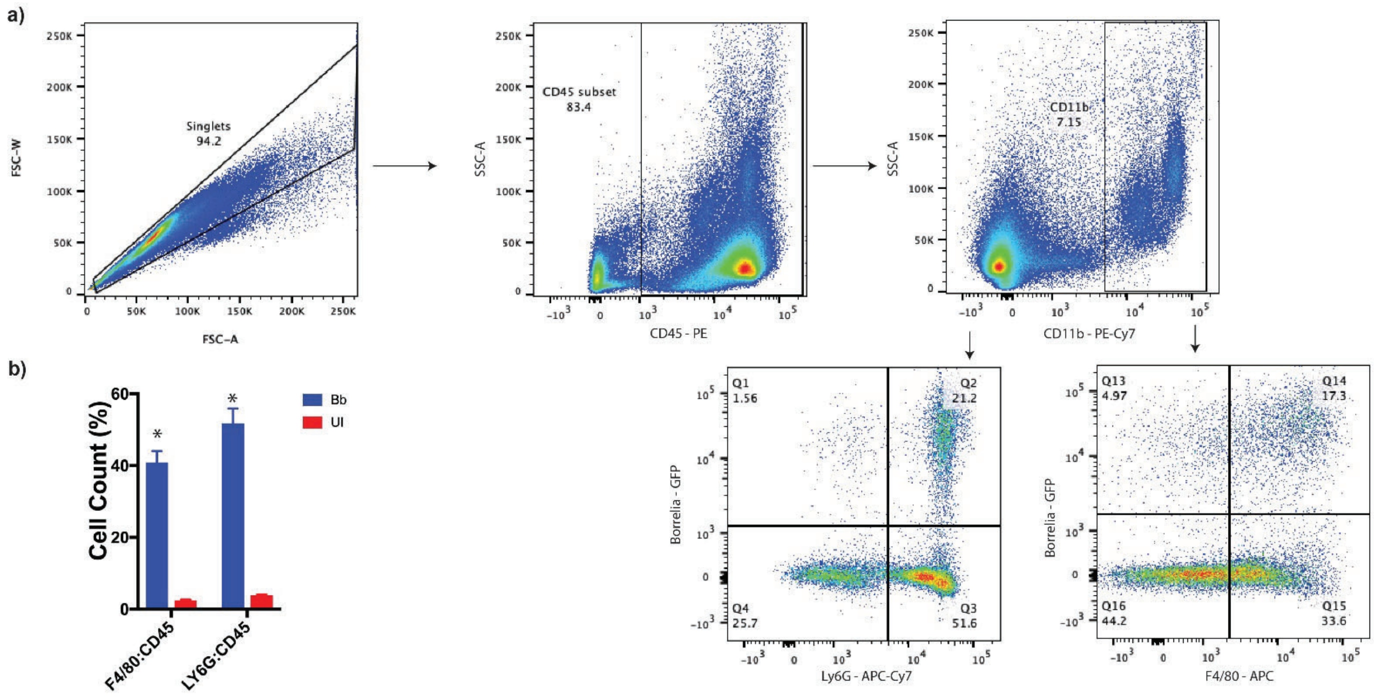

Supplementary Figure 4: Gene expression analysis of selected pro-inflammatory cytokines using RT-PCR comparing uninfected and *Bb* infected BMNs at 1,16 and 24 hpi represented as relative folds compared to the uninfected neutrophils at 1hpi (Second biological replicate).

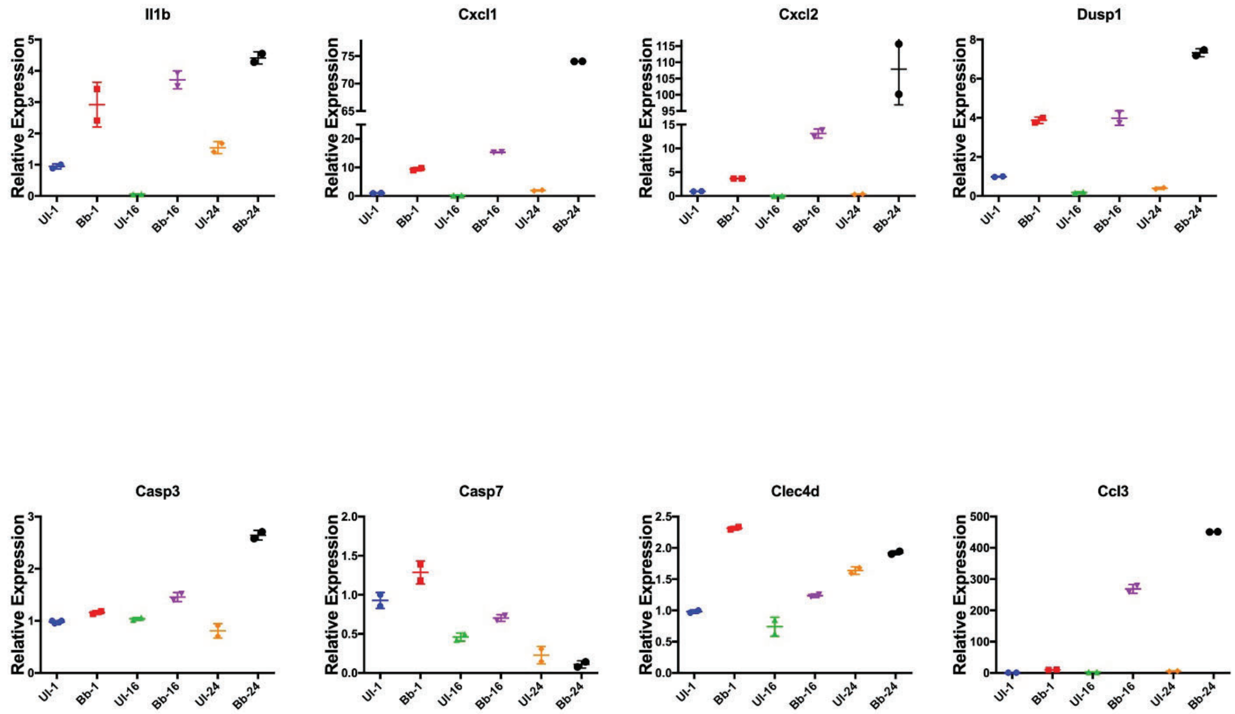

Supplementary Figure 5: Gene expression analysis of selected complement and cytokine encoding genes using RT-PCR analysis comparing uninfected and *Bb* infected BMDMs at a) 1, and b) 24 hpi (Second biological replicate).

a)

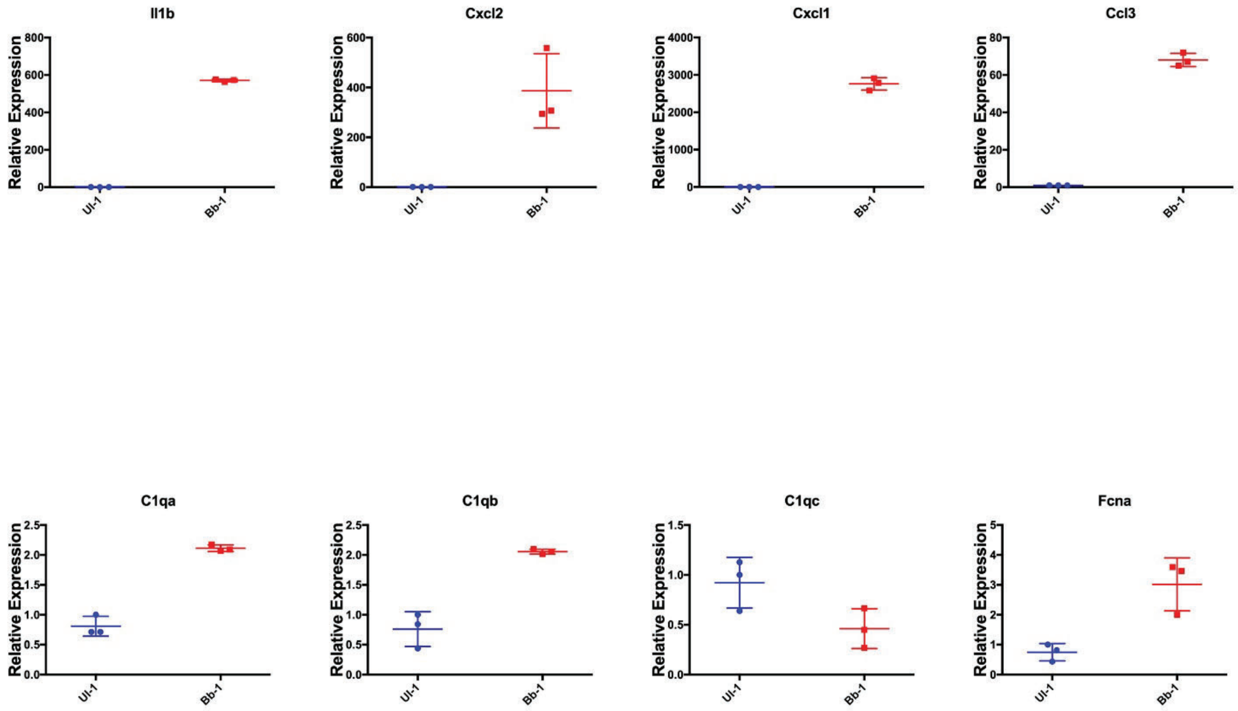

b)

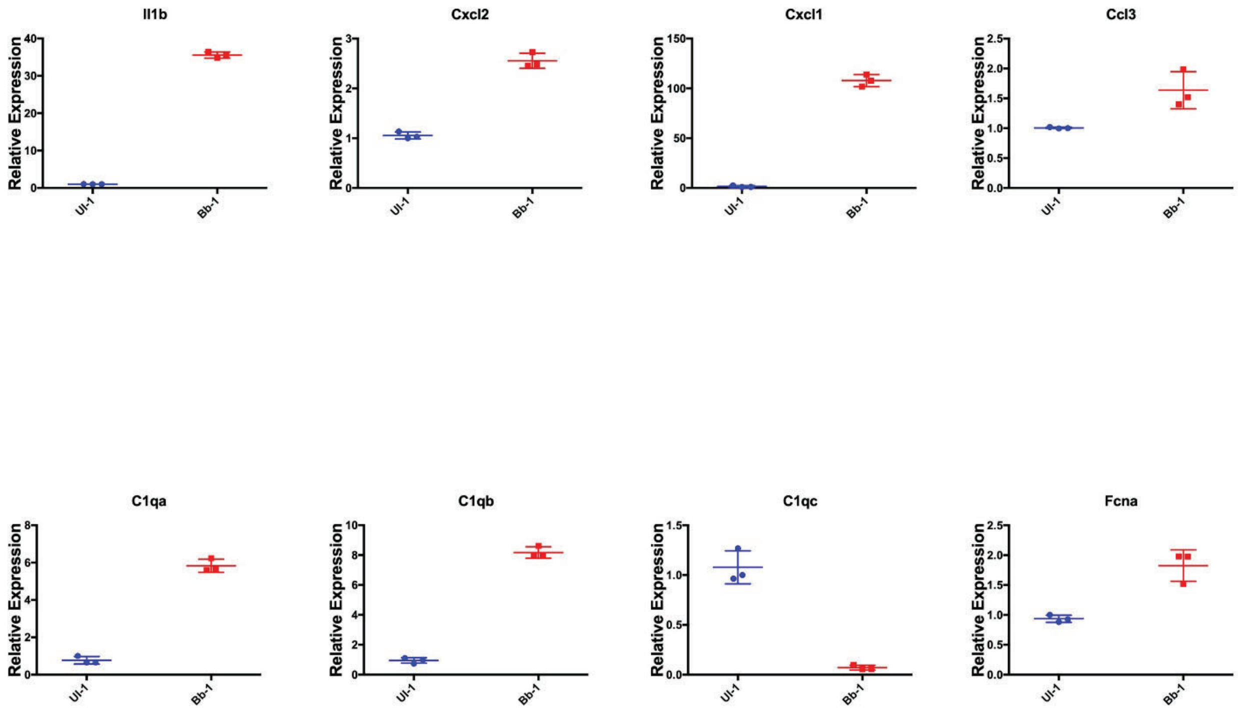

Supplementary Figure 6: Gene expression analysis of complement genes in BMDMs co-cultured with *Bb* infected BMNs at two different ratios of BMN:BMDM - 1:10 and 1:1. (Second biological replicate).

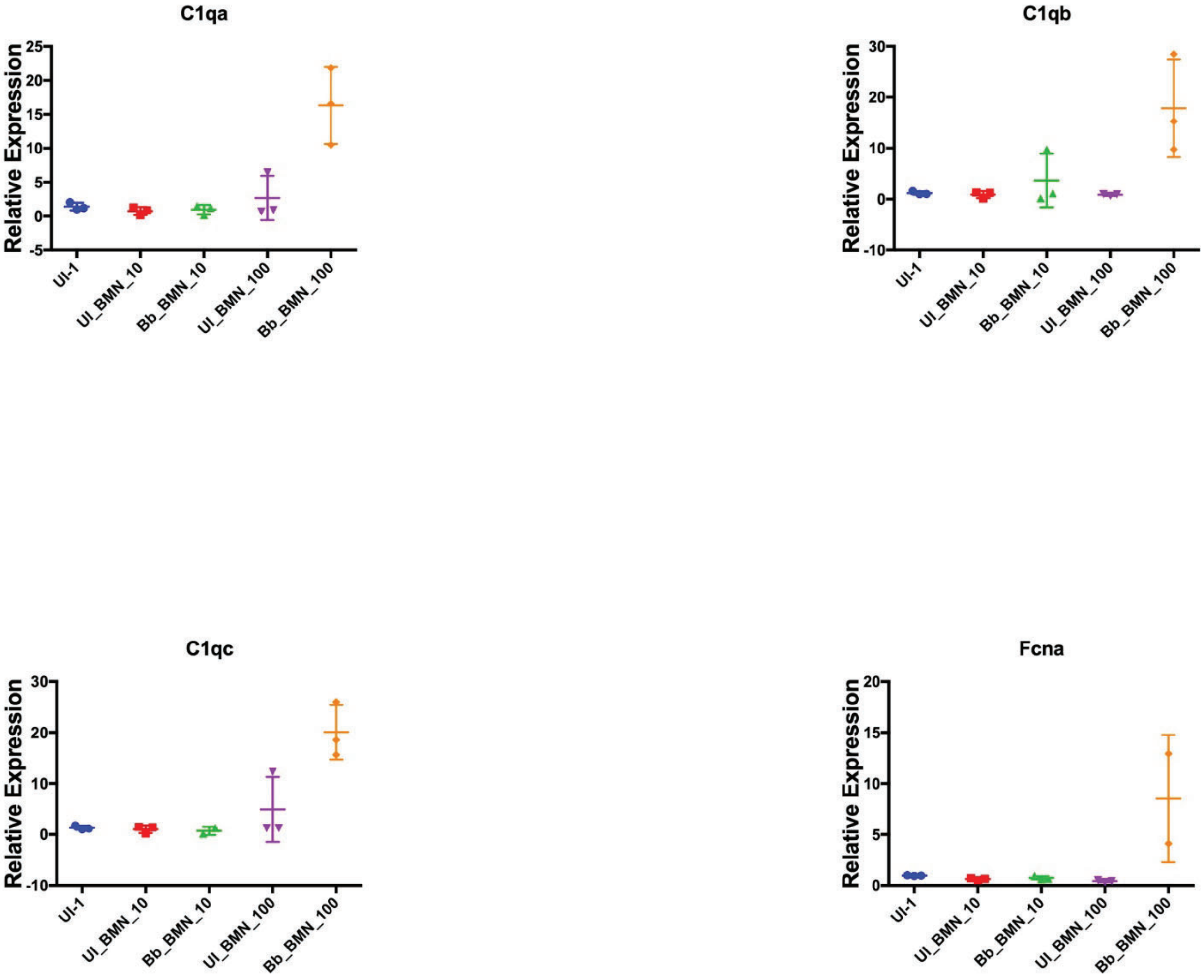

Supplementary Table 1: Primers used in this study

Actb-F	GAC TCA TCG TAC TCC TGC TTG
Actb-R	GAT TAC TGC TCT GGC TCC TAG
Cxcl2-F	CTT TCC AGG TCA GTT AGC CTT
Cxcl2-R	CAG AAG TCA TAG CCA CTC TCA AG
Cxcl1-F	GTG CCA TCA GAG CAG TCT
Cxcl1-R	CCA AAC CGA AGT CAT AGC CA
Ccl3-F	CGA TGA ATT GGC GTG GAA TC
Ccl3-R	CCT TGC TGT TCT TCT CTG TAC C
Dusp1-F	GAA CAA ACA CTC TCC CTC CAG
Dusp1-R	CAC TAC CAG TAC AAG AGC ATC C
Fcna-F	GTC TCC CAG CTC CTT TTC AC
Fcna-R	GCA GCC CAG GAA AGA TGG
C1qa-F	CAT CTT CAG CCA CTG TCC ATA
C1qa-R	ATC CAG TTT GAT CGG ACC AC
C1qb-F	CAG GAC ACA TGG AGA AAA CCT
C1qb-R	GCC TAG AAG CAT CAC AGA ACA
C1qc-F	TCG CCC TTC TGA CCC TT
C1qc-R	GCT GGC TGC TAT GGG AT
Clec4d-F	CAG TTT CAC CAC ACT TCC TCT
Clec4d-R	CTG ATC CCT TGC GTC TTC G
Casp7-F	TCC ATG CGG TAC AGA TAA GTG
Casp7-R	CAG CGA AGA CGG AGT TGA C
Casp3-F	GGA CTG GAT GAA CCA CGA C
Casp3-R	GAC TGA TGA GGA GAT GGC TTG
Il1b-F	CTC TTG TTG ATG TGC TGC TG
Il1b-R	GAC CTG TTC TTT GAA GTT GAC G
Ccl5-F	CCT CTA TCC TAG CTC ATC TCC A
Ccl5-R	GCT CCA ATC TTG CAG TCG T

Supplementary Table 2: Genes discussed in this study along with their respective protein ID, full name and human homologs

Gene ID	Protein ID	Full name	Human Homologs
ACOD1	IRG1	aconitate decarboxylase 1/Immune responsive gene 1	ACOD1
AHNAK	Desmoyokin	AHNAK nucleoprotein	AHNAK
ANKRD22	ANKRD22	ankyrin repeat domain 22	ANKRD22
APOE	APOE	apolipoprotein E	APOE
ATF3	ATF3	activating transcription factor 3	ATF3
C1QA	C1QD1	complement C1q A chain	C1QA
C1QB	C1QD2	complement C1q B chain	C1QB
C1QC	C1QD3	complement C1q C chain	C1QC
CASP3	CASP3	Caspase3	CASP3
CASP7	CASP7	Caspase7	CASP7
CCL3	MIP-1A	C-C motif chemokine ligand 3/Macrophage Inflammatory Protein-1 Alpha	CCL3
CCL5	RANTES	Regulated upon Activation, Normal T Cell Expressed and Presumably Secreted	CCL5
CD3d	CD3d	CD3 delta subunit of T-cell receptor complex	CD3d
CD3e	TCRE	CD3 epsilon subunit of T-cell receptor complex	CD3e
CD3g	CD3g	CD3 gamma subunit of T-cell receptor complex	CD3g
CD79a	IGA	immunoglobulin alpha	CD79a
CLEC4D	Dectin3	C-type lectin domain family 4 member D	CLEC4D
CLEC4E	Mincle	C-type lectin domain family 4 member E	CLEC4E
CSF1	MCSF	macrophage colony-stimulating factor	CSF1
CSF1R	CD115	colony stimulating factor 1 receptor	CSF1R
CTLA4	CD152	cytotoxic T-lymphocyte associated protein 4	CTLA4
CTSS	CTSS	Cathepsin S	CTSS
CXCL1	CXCL1	C-X-C motif chemokine ligand 1	CXCL1
CXCL2	MIP2	C-X-C motif chemokine ligand 2	CXCL2
DUSP1	MKP1	dual specificity phosphatase 1/mitogen-activated protein kinase phosphatase 1	DUSP1
F13A1	F13a	coagulation factor XIII A chain	F13A1
FAM214A	FAM214A	family with sequence similarity 214 member A	FAM214A
FCNA	Fcn1	FicolinA	FCN1
FN1	MSF	fibronectin 1/migration stimulating factor	FN1
Foxp3	Foxp3	forkhead box P3	Foxp3
H2-Aa	H2-Aa	histocompatibility 2, class II antigen A, alpha	HLA-DQA1
H2-Ab1	H2-Ab1	histocompatibility 2, class II antigen A, beta 1	HLA-DQB1
H2-Eb1	H2-Eb1	histocompatibility 2, class II antigen E, beta	HLA-DRB1
H2-Eb2	H2-Eb2	histocompatibility 2, class II antigen E, beta 2	

HCAR2	HCAR2	hydroxycarboxylic acid receptor 2	HCAR2
Hist1H1B	H1f5	H1.5 linker histone	Hist1H1B
Hist1H1D	H1f3	H1.3 linker histone	Hist1H1D
Hist1H3c	H3C3	H3 clustered histone 3	Hist1H3c
IGKC	IGKC	immunoglobulin kappa constant	IGKC
IGLC3	IGLC3	immunoglobulin lambda constant 3	IGLC3
Ikzf2	HELIOS	IKAROS family zinc finger 2	Ikzf2
IL17a	CTLA8	interleukin 17A/Cytotoxic T-Lymphocyte-Associated Serine Esterase 8	IL17a
IL17f	IL17f	interleukin 17F	IL17f
IL1B	IL1B	interleukin 1 beta	IL1B
IL1F9	IL36G	interleukin 36 gamma	IL1F9
IL7r	CD127	interleukin 7 receptor	IL7r
ITGAM	CD11b	Integrin alpha M	ITGAM
JCHAIN	IGJ	joining chain of multimeric IgA and IgM	JCHAIN
Klrc2	Klrc2	killer cell lectin like receptor C2	Klrc2
Lef1	Lef1	lymphoid enhancer binding factor 1	Lef1
Lilrb4a	CD85K	leukocyte immunoglobulin-like receptor, subfamily B, member 4A	LILRB4
Ly6c1	Ly6c1	lymphocyte antigen 6 family member C1	
Ly6c2	Ly6c2	lymphocyte antigen 6 family member C2	
Ly6G	Ly6G	Lymphocyte antigen 6 complex locus G6D	
Lyz2	Lys	lysozyme 2	LYZ
MMP8	MMP8	matrix metallopeptidase 8	MMP8
MMP9	MMP9	matrix metallopeptidase 9	MMP9
MYC	bHLHe39	MYC proto-oncogene, bHLH transcription factor	MYC
MZB1	MZB1	marginal zone B and B1 cell specific protein	MZB1
NR4A1	NR4A1	nuclear receptor subfamily 4 group A member 1	NR4A1
NR4A2	NR4A2	nuclear receptor subfamily 4 group A member 2	NR4A2
NR4A3	NR4A3	nuclear receptor subfamily 4 group A member 3	NR4A3
NUSAP	ANKT	nucleolar and spindle associated protein 1	NUSAP
Pafah1b3	PAFAHG	platelet activating factor acetylhydrolase 1b catalytic subunit 3	Pafah1b3
PDCD5	TFAR19	programmed cell death 5	PDCD5
PLSCR1	MMTRA1B	phospholipid scramblase 1	PLSCR1
PTPRC	CD45	protein tyrosine phosphatase receptor type c	PTPRC
RETNLG	RETNLG	resistin like gamma	
Rrp1b	NNP	ribosomal RNA processing 1B/Novel nuclear protein	Rrp1b

S100A4	FSP1	S100 calcium binding protein A4/Fibroblast-specific protein 1	S100A4
S100A6	s100a6	S100 calcium binding protein A6	S100A6
S100A8	MIF	S100 calcium binding protein A8/Macrophage migration inhibitory factor	S100A8
S100A9	MIF	S100 calcium binding protein A9	S100A9
SIGLECG	Siglec10	sialic acid binding Ig-like lectin G	SIGLEC10
SOD2	MnSOD	superoxide dismutase 2	SOD2
ST8SIA6	St8sia6	ST8 Alpha-N-Acetyl-Neuraminide Alpha-2,8-Sialyltransferase 6	ST8SIA6
SUCO	SUCO	SUN domain containing ossification factor	SUCO
Tcf7	tcf7	transcription factor 7	Tcf7
TDRP	Tdrp	testis development related protein	TDRP
TNFRSF4	CD134	TNF receptor superfamily member 4	TNFRSF4
TNFRSF9	CD137	TNF receptor superfamily member 9	TNFRSF9
TNFSF13B	BAFF	TNF superfamily member 13b/B cell activating factor	TNFSF13B
TRAC	Trac	T cell receptor alpha constant	TRAC
TRBC2	TCRBC2	T cell receptor beta constant 2	TRBC2
VPREB3	Vpreb3	V-set pre-B cell surrogate light chain 3	VPREB3
XBP1	TREB5	X-box binding protein 1	XBP1
ZFP36	ZFP36	ZFP36 ring finger protein	ZFP36

Table 3: Significant DEGs (p<0.01) between total splenocytes clusters:

T Lymphocytes (CD3e)			B Lymphocytes (CD19)										Myeloid (Csf1r)	NK cells (Ncr1)	Erythrocyte (Gypa)
C1	C7	C12	C2	C3	C4	C5	C6	C8	C11	C13	C14	C16	C15	C9	C10
Lef1	Rflnb	Icos	Srm	Gm31243	Gm31243	Dtx1	-	Apoe	Abca1	Ccl4	-	-	Lyz2	Gzma	Hba-a1
Rflnb	Lef1	Ikzf2	Nop16		Gm30211	Kcnq1ot1		Ly6d	Syk	Myc			S100a9	Ncr1	Alad
Itk	Gm12840	Tnfrsf4	Nhp2		Ighd			Siglecg	Mtss1	Rrp1b			S100a8	Klre1	Alas2
Tcf7	Cd3d	Rora	Timm8a1					Vpreb3		Irf4			Alox5ap	Cma1	Aqp1
Bcl11b	Il7r	Ctla4	Rrp1b					Cd79b		Nr4a3			Cd14	Klra3	Birc5
Dusp10	Skap1	Tnfrsf18	Nifk					Mtss1		Lilrb4a			Ngp	Eomes	Blvrb
Gm12840	Trac	Cd5	Bcl2a1b					Fcrla		Ccl3			Ifitm3	Serpinb9b	Bpgm
Il7r	Trbc2	Hopx	Gnl3					Cnp		Nop58			Camp	Ccl5	Car2
Trac	Tcf7	Ramp3	Bcl2a1d					Unc93b1		Srm			Hp	Sytl3	Cenpf
Rgs10	Itk	Cd3g	Tma16					Plac8		Tma16			Lcn2	Prf1	Cldn13
Cd3d	Ms4a6b	Il18r1	Nolc1					Syk		Znhit6			Ifitm6	Nkg7	Cpox
Trbc2	Rgs10	Cd3e	Marcksl1					Pax5		Nolc1			Ltf	Klri2	Ctse
Ms4a6b	Cd3g	S100a4	Ppan					Fcmr		Nr4a2			Clec4e	AW112010	Ermap
Cd3e	Saraf	Cd27	Myc					Erp29		Marcksl1			Chil3	Serpinb9	Fech
Cd28	Bcl11b	Rgs1	Hspd1					Ms4a1		Kdm6b			Ly6g	Klrk1	Gm26917
Skap1	Cd3e	Tbc1d4	Ndufaf4					Iglc3		Gnl3			Cxcl2	Spry2	Gpx1
Adk	Dusp10	Cd28	Odc1					Ptprcap		Ncl			Ptgs2	Serpinb6b	Gypa
Trbc1	Trbc1	Emb	Nop58					Pold4		Bcl2a1d			Anxa1	Il12rb2	Hba-a2
Cd3g	Emb	Rab8b	C1qbp					Ly6e		Mat2a			Thbs1	Klrc2	Hbb-bs
Fyb	Cd28	Bcl11b	Tomm5					Dnajc7		Nifk			Wfdc21	Il18rap	Hbb-bt
Emb	Ms4a4b	Trbc2	Snhg15					Ifi30		Nop16			Il1b	Fasl	Hemgn
Saraf	Gtf2i	Rnf125	Mif							M6pr			Siglech	Klrd1	Hmbs
Kif1b	Ramp3	Trbc1	Snhg4							Pik3ap1			Lst1	Klrc1	Isg20
Stat5b	Adk	Ctla2a	Ddx21							Egr3			Ifitm2	Gzmb	Mgst3
Grap2	Rnf125	Parp8	Irf4							Snhg15			Pglyrp1	Il2rb	Mki67
Ms4a4b	Kif1b	Trac	Ncl							Hspd1			Ier3	Ccr5	Nusap1
Cd5	Grap2	Fyb	Dkc1							Bcl2a1b			Ly6c2	Ctsw	Pdzk1ip1
Tbc1d4	Fyb	Klrc1	Atad3a							Slc38a1			Lgals3	Ctla2b	Prc1
Zeb1	Txk	Ctla2b	Lyar							Gch1			Retnlg	Cemip2	Prdx2
Ramp3	Npc2	Tnfaip3	Znhit6							Plek			Mpeg1	Ctla2a	Rhd

Dgka	Cd27	Cd3d	Nsun2							Ppan			Fcer1g	Pde2a	Rrm2
Gtf2i		Lgals1	Pgam1							Dkc1			Ccr9	Il18r1	Rsad2
Rnf125		Klrc2	Eif1ad							Wdr43			Jchain	Ccr2	Slc25a37
Cd27		Pik3r1	Wdr43							Atad3a			Plbd1	Anxa2	Slc4a1
Txk		Ccr2	Sdad1							Tank			Basp1	Prex1	Slfn14
Smc4		Fasl	Pa2g4							Nr4a1			Csf2rb	Sh2d2a	Snca
Tmem64		Il2rb	Rilpl2							Atf3			Fos	Irf8	Spta1
Vps37b		Ctsw	Tomm40							Odc1			S100a6	Parp8	Tfdp2
S1pr1		Dusp10	Rsl1d1							Nop56			Dstn	Zyx	Tmcc2
Spn		Fosl2	Ran							Swap70			Ctsl	Pik3r1	Top2a
Crlf3		Skap1	Ranbp1							Nfkbid			S100a4	Txk	Trim10
Gimap3		Ifngr1	Npm1							Nsun2			Bst2	Gm19585	Tspan33
Gramd3		Tmem64	Mrpl12							Eif4e			Tyrobp	Lgals1	Ube2c
Tnfrsf18		Hcst	Cct3							Ndufaf4			Cdkn1a	Ifngr1	Ube2l6
Gimap4		Rgs2	Plek							Pdcd11			Igkc	Ugcg	
Lck		Sh2d2a	Anp32b							Nhp2			Msrb1	Rgs1	
Crbn		Itpkb	Timm23							Tfrc			Ms4a6c	Nabp1	
Stk38		Pde2a	Slc7a5							Timm8a1			Anxa2	Tyrobp	
Klhl6		Ifi27l2a	Cd86							Slc7a1			Anxa5	Ctsd	
Cblb										Ybx3			Cst3		

Table 4: Significant DEGs (p<0.01)

a) between overall neutrophils clusters:

Inflammatory Neutrophils	APC Neutrophils	Apoptotic Neutrophils	NETosis Neutrophils
**C1**	**C2**	**C3**	**C4**
Inhba	Jchain	Cstdc4	Asf1b
Plscr1	Iglc2	Ccl6	Brca2
Zmpste24	Ighg2b	Ccl3	Ccne1
Orm1	Igha	Ccrl2	Ccne2
Tnfsf13b	Igkc	Cxcl2	Ect2
Mgst2	Mzb1	Fosl1	Esco2
Tomm70a	Ighm	Hcar2	Fbxo5
Olr1	Ms4a1	Mirt2	Gm10457
Ankrd22	Igkv14-111	Fpr1	Gmnn
St3gal5	Ighv1-82	Asprv1	Mybl2
Slco4c1	Igkv8-27	Spp1	Nuf2
Rcor1	Iglc3	Ifitm1	Nusap1
Tex15	Cd74	Clec4d	Pbk
Ldhc	Ebf1	Slc7a11	Pola1
Cpne3	H2-Ab1	Slc15a3	Prc1
Rhou	Iglc1	Osm	Rad51ap1
AA467197	H2-Aa	Gpr27	Rrm2
Slc31a2	Txndc5	Egr1	Sgo1
Tmem216	Hmgn1	Slc16a3	Wdr76
Megf9	C1qbp	Ccr1	Hist1h1b
Sort1	Pou2af1	Cd300lb	Hist1h1d
Tmem45a2	Pou2f2	Ier3	Hist1h2ab
Iqgap2	Gm30211	Bmx	Hist1h2ae
	Tnfrsf13c	Nlrp3	Hist1h2af
	Fkbp11	Stfa1	Hist1h2ap
	Ctss	Stfa2	Hist1h2bl
	Tnfrsf17	Stfa2l1	Hist1h2bn
	Iglv1	Retnlg	Hist1h3c
	Gpr183		Hist1h3e

b) between infected and bystander neutrophils

Inflammatory Neutrophils	APC Neutrophils	Apoptotic Neutrophils	NETosis Neutrophils
**C1-Bb**	**C2-Bb**	**C3-Bb**	**C4-Bb**
Ccl5	Ighg2b	Il1b	Birc5
Sod2	Cxcl2	Ccl4	Ccdc34
mt-Nd3	Slpi	Dusp1	Cd3d
	Olfm4	Ccrl2	Cdca8
	Iglc2	Fosb	Cenpe
	Igkv8-27	Cstdc4	Eif3c
	Stfa3	Clec4d	Esco2
	Mt1	Asprv1	Flot2
	Ighv1-82	Dennd4a	Kif15
	Il1b	Hcar2	Nusap1
		Ccl3	Rpa3
		Stfa1	Rrm2
		Gm26740	Serinc3
		Tubb4b	Spc25
		Cxcl2	Tuba4a
		Spp1	Ucp2
		Entpd1	Hist1h1b
		Fos	Hist1h2ap
		Junb	Hist1h3c
		Ppp1r10	Hist1h2ae
		Mindy1	Hist1h1d
		Ifitm1	Hist1h1c
		Emb	
		Osm	
		Ifi27l2a	

Table 5: Significant DEGs (p<0.01)

a) between overall macrophages clusters:

C1	C2	C3	C4	C5
Ebf1	Ly6c2	C1qb	Ace	Cd209a
Cd79a	Ctsl	Il1rn	Gngt2	Zbtb18
Igkc	F13a1	C1qa	Fcgr4	Flt3
Ms4a1	Lyz2	Itgad	Pltp	Tep1
Gnl3	Ly6c1	Fcna	Adgre4	Timd4
Tnfrsf13c	Prdx5	S100a9	Itgal	Syngr2
Cd79b	Gstm1	Axl	Spn	Gcnt2
Ighd	Thbs1	C1qc	Dgkh	Slco3a1
Ighm	Msr1	Vcam1	Cd36	Cd7
Mzb1	Mgst1	AW011738	Cd300c2	P2ry10
Scd1	Cd177	S100a8	Pou2f2	Vps37b
Fcmr	Fn1	Hdc	Eno3	Iqgap2
Cr2	Vcan	Pilra	Fyb	Klrk1
Bank1	Gda	Ccl3	Sgk1	Ramp3
Irf4	C3	Wfdc21	Cyth3	Csf2rb2
Dtx1	Ifitm3	Ccl4	Pdgfb	Cd209d
Dkc1	Arsb	Ctsd	Olr1	Fem1c
Iglc2	Ctsc	Trem3	Bcl2a1a	Cenpa
Cd72	Cxcl1	AW112010	Lima1	Abcb1b
Myo1e	Chil3	Entpd1	Tgm2	Eps8
Blk	AI839979		Hip1	Jak2
Fcer2a	Gpr141		Abcg1	Csf2rb
Ncl	Serpinb8		Fgr	Lgals1
Satb1	Cd93		Fabp4	Rab43
Ndufaf4	Clec4a2		Clec4a3	Il7r
Dmxl1	Grk5		Heg1	Rora
Nolc1	Emp1		Dusp16	Cmtr1
H2-Ob	Il6		Treml4	Dock10
Wdr43	Ifitm6		Pglyrp1	Il2rb
Ppp1r16b	Ldlr		Slc12a2	H2-DMb1
Npm1	Prdm1		Nupr1	Il22
Nop56	Npc2		Tnfrsf1b	Zfp263
Phgdh	Ptgs2		Plxnb2	H2-Aa
C1qbp	F10		Ear2	Gatad1
Cd37	Smpdl3a		Stk10	Pid1
Alkbh1	Tlr2		Atp1a1	Gfpt1
Nifk	Ccr2		Lair1	Tuba1a
Hmgn1	Ms4a6c		Itgb2	Tnip3
Nop58	Anxa1		Il10ra	Tbc1d4
Nhp2	Avpi1		Slc8b1	Cir1
Atad3a	Frrs1		Sec14l1	Tut4
Nop16	Evi2a		Lipa	Ywhah
Ralgps2	Lgals3		Adcy7	Isy1
Srm	Atp2b1		Cx3cr1	St3gal5

b) between infected and bystander cells

C3-Bb
S100a9
S100a8
Fcna
C1qc
C1qb
C1qa
Il1b
Ccl4

Table 6: Significant DEGs (p<0.01) between B cell clusters

				Precursor B cell	T cell interacting GC B cell		Marginal zone B cell	CD3+ B cells	Antibody secreting B cells
**C1**	**C2**	**C3**	**C4**	**C5**	**C6**	**C7**	**C8**	**C9**	**C10**
Neurl3	-	Nop16	Sell	Akap12	**Nr4a3**	-	Apoe	Itk	Igkc
		Timm8a1		Pafah1b3	Myc		Pafah1b3	Trbc2	Cacna1s
		Srm		Blvrb	Lilrb4a		Ahnak	**Cd3e**	**Ighm**
		Nhp2		Car2	**Nr4a2**		**Mzb1**	Tcf7	Fam214a
		Bcl2a1d		**Vpreb3**	Irf4		Ly6d	Fyb	Xbp1
		St6galnac4		Iglc3	Rrp1b		Plac8	Ccl5	Igkv1-117
		Rrp1b		Iglc1	Tma16		Igha	Cd3d	Sdf2l1
		Bcl2a1b		Prdx2	Kdm6b		Pdia4	Ms4a4b	Ighv6-6
		Marcksl1		Iglc2	Bcl2a1d		Zbtb20	Il2rb	Gm26917
		Samsn1		Ltb	Ccl4		Crip1	Il7r	Ighv6-3
		Ppat		Slfn2	Nifk		Aldh2	Cd3g	Ly6c2
				Fchsd2	Eif4e		Dnajc7	Trbc1	Jchain
				Atpif1	M6pr		Txndc5	Ctla2a	Eaf2
				**Siglecg**	Nop58		Sec11c	AW112010	Creld2
				Ptprcap	Srm		Lsp1	Nkg7	Edem2
				Arpc5l	Ccl3		Iglv1	Gzma	Txndc5
				Fcer2a	Mat2a		Erp29	Txk	Igkv9-120
					Bcl2a1b		Mtss1	Emb	Iglv1
					Tfrc		Npc2	Rflnb	Igkv8-27
					Znhit6		Stk38	Lef1	Fkbp2
					Samsn1		Unc93b1	Gm12840	Sel1l
					Bzw2		Eaf2	Ifngr1	Prdx4
					Nolc1		Lyz2	Rgs1	
					Snhg15			Ms4a6b	
					Gnl3			Saraf	
					Ncl			Tnfaip3	
					Ppan			Pik3r1	
					St6galnac4			Vps37b	
					Tank			Fxyd5	

Table 7: Significant DEGs (p<0.01) between T cell clusters

		Non-lymphoid T cell		CD3+ B cells	NK cells	CD8 T cell
**C1**	**C2**	**C3**	**C4**	**C5**	**C6**	**C7**
**Cd4**	**Cd4**	**Cd4**	**Cd4**	**Cd4**	Ccl5	**Cd8a**
	St8sia6	Tnfrsf4	St8sia6	Ebf1	**Klrc2**	Cd8b1
		Ctla4		Mef2c	Klrk1	Klrd1
		S100a4		Ms4a1	Nkg7	
		Tnfrsf9		H2-Aa	Gzma	
		Ikzf2		Cd79a	Trdc	
		Icos		Cd74	Cxcr6	
		Hopx		H2-Eb1	Serpinb6b	
		Maf		Fcmr	Cd7	
		Tnfsf8		H2-Ab1	Pdcd1	
		Smco4		Cd79b	Parp8	
		Sla		Ifi30	AW112010	
		Lmnb1		Iglc2	Il2rb	
		S100a6		Ighd	Klrd1	
		Rora		Tcf4	Dusp5	
		Itgb1		Mzb1	Abcb1a	
		Lgals1		Lyn	Samsn1	
		Eea1		Iglc1	Irf8	
		Cd5		Plek	Nr4a2	
		Tnfrsf18		Ctsz	Fosl2	
		Faim		Myo1e	Ctla2a	
		Tnfaip8		Stap1	Pde2a	
		Tbc1d4		Ighm	Pik3r1	
		Rgs1		Swap70	Id2	
		Ifi27l2a		Igkc	Ybx3	
		Tox		Plac8	Rgs1	
		Hif1a		Rilpl2	Stat3	
		Got1		Pou2f2	St8sia4	
		Cd2		Rel	D16Ertd472e	
		Trat1		Cst3	Ahnak	
		Ramp3		Nr4a1	Rgs2	
		Rab8b		Gnl3	Neurl3	
		Rgs2		Irf8	Rora	
		Jak2		Ly6e	Bcl2l11	
		Cd6		Dmxl1	Bcl2a1b	
		Gna13		Jchain	Dennd4a	
		Prr13		Kcnq1ot1	Rab8b	
		Samsn1		Snx5	Sh2d2a	
		Nr3c1		Lars	Zfp36l2	
		Ahnak		Slc38a1	Crem	
		Vim		C1qbp	Ostf1	
		Crip1		Nars		
		Slc4a7		Dusp2		
		Bcl2a1b		Nop58		
